# The Tissue-Associated Microbiota in Colorectal Cancer: A Systematic Review

**DOI:** 10.3390/cancers14143385

**Published:** 2022-07-12

**Authors:** Carolina Pinto da Costa, Patrícia Vieira, Melissa Mendes-Rocha, Joana Pereira-Marques, Rui Manuel Ferreira, Ceu Figueiredo

**Affiliations:** 1 Instituto de Investigação e Inovação em Saúde, Universidade do Porto (i3S), 4200-135 Porto, Portugal; carolinapintodacosta@gmail.com (C.P.d.C.); pvieira@ipatimup.pt (P.V.); mrocha@ipatimup.pt (M.M.-R.); jmarques@ipatimup.pt (J.P.-M.); ruif@ipatimup.pt (R.M.F.); 2 Institute of Molecular Pathology and Immunology of the University of Porto (IPATIMUP), 4200-135 Porto, Portugal; 3 Department of Pathology, Faculty of Medicine of the University of Porto, 4200-319 Porto, Portugal

**Keywords:** mucosal microbiota, colorectal cancer, microbiome, bacteria, 16S rRNA sequencing, next-generation sequencing

## Abstract

**Simple Summary:**

Growing evidence shows a close relationship between the microbiome and colorectal cancer, but most studies analyze fecal samples. However, solid information on the microbial community that is present locally in the intestinal tumor tissues is lacking. Therefore, the aim of this systematic review was to compile evidence on the relationship between tissue-associated microbiota and colorectal cancer. Among 5080 screened publications, 39 were eligible and included in the analysis. Despite the heterogeneity in methodologies and reporting between studies, 12 groups of bacteria with strong positive and 18 groups of bacteria with strong negative associations with colorectal cancer were identified. Such knowledge may ultimately be used in novel strategies that aim to prevent, detect, and treat colorectal cancer in the upcoming years.

**Abstract:**

The intestinal microbiome is associated with colorectal cancer. Although the mucosal microbiota better represents an individual’s local microbiome, studies on the colorectal cancer microbiota mainly reflect knowledge obtained from fecal samples. This systematic review aimed to summarize the current evidence on the relationship between the mucosal-associated bacterial microbiota and colorectal cancer. Searches were conducted in PubMed and Web of Science databases for publications comparing the mucosal microbiome of colorectal cancer patients with that of healthy controls, or with that of non-cancerous mucosal tissues. The primary outcomes were differences in microbial diversity and taxonomy. The Newcastle-Ottawa Scale was used to assess the quality of the included studies. Of the 5080 studies identified, 39 were eligible and included in the systematic review. No consistent results were identified for the α- and β-diversity, due to high heterogeneity in reporting and to differences in metrics and statistical approaches, limiting study comparability. Qualitative synthesis of microbial taxonomy identified 12 taxa with strong positive and 18 taxa with strong negative associations with colorectal cancer. *Fusobacterium*, *Campylobacter*, *Parvimonas*, *Peptostreptococcus*, *Streptococcus*, and *Granulicatella* were defined as enriched in colorectal cancer. Despite the methodological limitations of the studies, consistent evidence on bacterial taxa associated with colorectal cancer was identified. Prospective studies in large and well-characterized patient populations will be crucial to validate these findings.

## 1. Introduction

Colorectal cancer (CRC) is the third most frequent cancer and the second leading cause of death due to cancer, for men and women, in the world [1,2]. Despite the implementation of CRC screening programs aimed at reducing cancer incidence and mortality, a significant proportion of cases are still diagnosed at advanced stages [3]. While early-stage CRC patients usually have a good prognosis, and curative surgical control of the disease is possible, patients with metastatic disease have a five-year survival rate of 14% [4,5]. In the latter, radiotherapy and chemotherapy are the leading strategies for controlling disease, and targeted therapy approaches have also been successful in prolonging the overall survival of CRC patients [3,5].

The great majority of CRC cases are sporadic (70% to 80%), a subset have a hereditary component, and another subset may occur as a consequence of inflammatory bowel diseases [6]. Thus, CRC is considered as a complex disease resulting from the interactions of environmental and genetic risk factors, leading to the accumulation of genetic alterations that dysregulate oncogenic and tumor suppressor signaling pathways [7,8].

The human body is inhabited by large communities of microorganisms–the microbiota–that together with their genome and the niche with which they interact constitute the microbiome [9]. The microbiome plays an important role in the normal human physiology, and alterations to the microbiome–host homeostasis, also known as dysbiosis, can affect the development and progression of several diseases, including cancer [10]. Increasing evidence supports the hypothesis that local dysbiosis contributes to carcinogenesis, by stimulating inflammation, cell proliferation, and even direct DNA damage [11,12,13]. In fact, mechanistic evidence has been provided for the involvement of *Fusobacterium nucleatum*, *Bacteroides fragilis*, and colibactin-producing *Escherichia coli* in the pathogenesis of CRC [14,15,16]. Furthermore, patients with CRC have distinct bacterial colonization patterns in their tumor tissues in comparison with their non-neoplastic mucosa or with the mucosa of healthy subjects [17,18,19].

A large number of studies that address the CRC microbial community composition have used fecal samples, prompted by their potential use as a non-invasive tool for cancer screening and early detection [20,21]. In fact, recent data collected in a national screening program suggest that microbial profiling may improve CRC screening accuracy [22]. However, while the use of fecal samples for microbiome studies may have advantages due to their ease of collection and non-invasive nature, their composition does not accurately represent the more stable cancer microbiota and the mucosal interactions across the gut [23,24]. The local tissue microbial community has a central role in colorectal chronic inflammation and tumorigenesis, and knowledge on its features is critical [25].

Since previous systematic reviews have explored the association of CRC with the microbiome, focusing mainly on the fecal microbiome [26,27] or on specific species such as *Fusobacterium nucleatum* [28,29], the aim of the present systematic review was to summarize the current evidence on the relationship between the mucosal microbiome and CRC. We reviewed the studies comparing the microbiota of patients diagnosed with CRC with that of healthy control individuals, and the studies comparing the microbiota in the cancer and in the non-cancerous mucosal tissues of CRC patients.

## 2. Materials and Methods

A systematic review was undertaken with the aim of identifying peer-reviewed publications that address the mucosal microbiome based on 16S rRNA gene characterization by next-generation sequencing and CRC. The systematic review followed the recommendations of the Preferred Reporting Items for Systematic Reviews and Meta-Analyses (PRISMA) [30]. The protocol has not been registered.

### 2.1. Eligibility Criteria

The inclusion criteria were: original studies comparing the human colonic tissue microbiota of patients with a confirmed diagnosis of CRC with that of healthy controls, or with the non-cancerous adjacent tissue. Only studies evaluating the microbiota by next-generation sequencing of the 16S rRNA gene performed in fresh/frozen tissue were included. Studies not written in English, reviews, opinion articles, letters, conference reports or abstracts, studies without a comparison group, studies performed in paraffin-embedded tissues, studies in animals, and in vitro studies were excluded. Studies addressing only precancerous lesions and studies targeting only specific microbes (e.g., *Fusobacterium nucleatum*) were also excluded.

### 2.2. Information Sources and Search Strategy

Searches were conducted in the PubMed and Web of Science (WoS) databases. For PubMed, the search strategy used Medical Subject Headings (MESH), as follows: (((microbiota[MeSH Terms]) OR (human microbiome[MeSH Terms]) OR (microbiome[MeSH Terms])) AND ((cancer, colorectal[MeSH Terms]) OR (carcinoma, colorectal[MeSH Terms]) OR (colorectal neoplasms[MeSH Terms]) OR (colon cancer[MeSH Terms]) OR (colon neoplasms[MeSH Terms]) OR (rectal cancer[MeSH Terms]) OR (rectal neoplasms[MeSH Terms]) OR (gastrointestinal cancer[MeSH Terms]) OR (gastrointestinal neoplasms[MeSH Terms]))), from inception through 31 December 2021. For the WoS database, we searched the Science Citation Index Expanded from inception up to 2021, and the search strategy was the combination of #1 TS = (microbiota OR microbiome) AND #2 TS = (colorectal neoplasm OR colorectal cancer OR colon cancer).

### 2.3. Selection Process

Two authors (CPC and PV or MM-R) independently reviewed studies retrieved by the search strategies and excluded studies based on titles and/or abstracts. When there was no consensus, the study was maintained for full text analysis. The same authors independently reviewed the selected studies for full text analysis. When there were discrepancies between reviewers, there was a re-check of data followed by a discussion to reach consensus, arbitrated by the senior author (CF).

### 2.4. Data Collection Process and Data Items

Two authors (CPC and PV or MM-R) independently extracted the great majority of the data, with the exception of the α- and β-diversity parameters and the statistical methods for microbiota features, which were extracted by JP-M and RMF. Discrepancies between reviewers were arbitrated by CF. The primary outcomes were differences in microbial α- and β-diversity, and in microbial taxonomy between tumor and healthy or non-cancerous tissue. Only taxa with statistically significant differences at a *p* value < 0.05 were considered. The following additional data from the included studies were also collected: country, number of participants, gender, age group, details of recruitment and intervention, and details on the microbiota characterization methods, including the targeted 16S rRNA region, the sequencing platform, the database for taxonomy assignment, the parameters to determine the α- and β-diversity, and the statistical methods used for comparisons of the microbiota parameters.

### 2.5. Methodological Quality

The quality of the included studies was assessed using the Newcastle–Ottawa Scale (NOS) [31]. The full evaluation score was 9 points, and comprised: (1) the selection of study population, including case definition, case representativeness, control selection, and control definition; (2) the comparability of the study groups, including the control for age and the control for other relevant confounders; and (3) the ascertainment of the outcome of interest, including the use of antibiotics as an exclusion criterion, the same method of ascertainment for cases and controls, and the use of clearly described statistics to analyze the data. A high-quality study was defined as having at least 7 points. Two reviewers (CPC, PV, or MM-R) assessed the quality of the included studies for selection, comparability, and ascertainment of the outcome of interest. CPC performed consensus. JP-M and RMF assessed the study quality for the use of statistics for data analysis.

### 2.6. Qualitative Synthesis of Microbial Taxonomy Results

For summarizing the microbial taxonomic relationships with CRC, qualitative synthesis was performed. When ≥3 studies detected a specific taxon statistically significantly enriched in CRC, and none identified it enriched in the healthy control or in the non-cancerous mucosa, the association was considered as strongly positive. Conversely, when ≥3 studies detected a specific taxon enriched in the mucosa of healthy controls or in the non-cancerous mucosa, and none identified it enriched in CRC, the association was considered as strongly negative. The associations were considered as suggestive when only two studies identified statistically significantly associations in the same direction and no studies identified associations in the opposite direction [32].

## 3. Results

### 3.1. Literature Search and Selection of Eligible Studies

**Figure 1:**
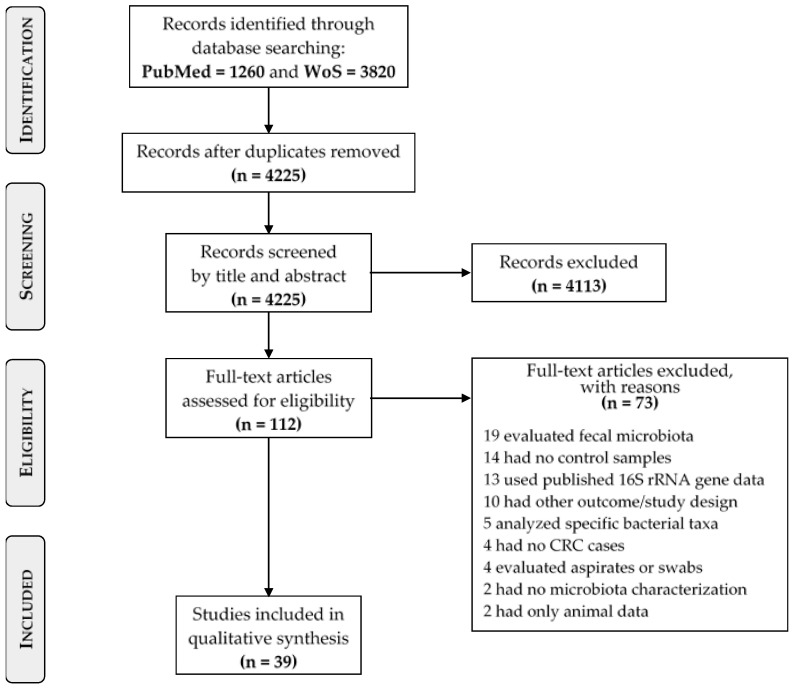
PRISMA flow-chart of the study selection process.

The initial literature search yielded a total of 5080 studies. Removal of duplicates resulted in 4225 articles, which were screened for eligibility based on title and abstract. After assessing the full text of 112 studies, 73 studies were additionally excluded as not meeting the requirements: 19 evaluated only fecal microbiota; 14 did not include controls; 13 used 16S rRNA data from previously published studies; 10 had another outcome or study design; five analyzed specific bacterial species, four did not have CRC cases; four used colonoscopy aspirates or tissue swabs; two did not characterize the microbiota; and two presented animal data only. A total of 39 studies were included in the present systematic review [19,33,34,35,36,37,38,39,40,41,42,43,44,45,46,47,48,49,50,51,52,53,54,55,56,57,58,59,60,61,62,63,64,65,66,67,68,69,70]. The PRISMA flow-chart of the study inclusion process is shown in Figure 1.

### 3.2. Population Characteristics and Quality Assessment of the Included Studies

An overview of the 39 included studies, their study design, and methods are summarized in Table 1 and Table 2, and in Supplementary Tables S1 and S2. Studies were published between 2011 and 2021, the majority (28 of 39) having been published in the last 5 years. Studies were conducted in Europe (n = 11), North America (n = 4), South America (n = 2), Asia (n = 20), and Australia (n = 1). One study included patients from both Europe and North America [37].

The tissue microbiota composition of CRC was compared with that of healthy controls in six studies [33,34,35,36,68,69] (Table 1) and with that of non-cancerous colon tissue of CRC patients in 27 studies (Table 2) [19,37,38,39,40,41,42,43,44,45,46,47,48,49,50,51,52,53,54,55,56,62,63,64,66,67,70]. Six studies included both comparisons [57,58,59,60,61,65]. The number of participants used for microbiome analysis ranged between 6 [19,55] and 115 [57], with 16 studies analyzing ≤ 25 participants (Table 1 and Table 2) [19,33,34,36,37,38,42,43,45,47,48,49,50,51,55,63]. The small sample size in these studies may reduce the accuracy of the results. In studies comparing CRC patients with healthy controls, CRC patients tended to be older than controls (Table 1), with only one exception [58]. In studies comparing the tumor tissue and the non-cancerous tissue of CRC patients, the localization and distance from the tumor where the non-cancerous tissue was collected showed large variation (Table 2).

All included studies used 16S rRNA gene sequencing for microbiome analysis, with the great majority targeting variable regions V3 and V4, alone or in combination with other regions, three targeting the V1–V2 regions [33,37,45], one targeting the V5-V6 regions [39], one targeting the V6 region [36], and two not reporting the target region [67,69]. The most frequently used sequencing platforms were Illumina MiSeq and 454 GS FLX (Roche). Studies resorted to different databases for taxonomic assignment, with Greengenes, SILVA, and Ribosomal Database Project (RDP) databases being the most frequently used. For statistical analyses of the taxonomic differences between the groups, seven studies did not report the methods used [19,34,38,43,47,49,58], whereas the remaining studies used distinct statistical approaches to analyze the data (Supplementary Tables S1 and S2).

The quality assessment of the included studies using the NOS scoring system is summarized in Supplementary Tables S3 and S4. Studies scoring ≥ 7 were considered as having high quality. Of the 12 studies comparing the colorectal microbiome in CRC and in healthy controls, seven [34,35,57,58,59,60,68] had a high-quality score of ≥7, with the median score being 7 (range 3–8). Of the 33 studies comparing tumors vs. non-cancerous mucosa, 27 [37,39,40,42,44,46,47,48,50,51,52,53,54,55,56,57,58,59,60,61,62,63,64,65,66,70] scored ≥7, with the median score being 7 (range 6–9). In the criterion selection, and in all included studies, the most frequent problem identified was the lack of information about the case representativeness of the population. In comparability, while all studies comparing samples of tumor and non-cancerous mucosa of CRC patients had the maximum score, only six studies [34,35,57,58,59,68] comparing CRC and healthy controls had the maximum score, with some studies not performing correction for confounders between cases and controls, allowing other factors to have a possible impact on results and reducing comparability. Regarding the ascertainment of the outcome, 22 (56.4%) [19,33,34,37,38,39,41,42,43,45,46,48,49,50,55,56,59,61,62,63,67,69] of all included studies did not include the use of antibiotics as an exclusion criterion (Supplementary Tables S3 and S4). In addition, 17 studies did not perform or report the statistical methods used for taxonomic and/or diversity comparisons [19,34,36,38,41,42,44,45,46,47,49,52,53,58,68,69,70]. Because of the variation in different aspects of the studies, the ability to summarize the results and conclusions and to compare individual results limited to a certain extent.

### 3.3. Microbial Diversity Findings

The main findings of the microbial α-diversity (i.e., within sample diversity) and β-diversity (i.e., diversity between samples) are detailed in Table 3 and Table 4. Diversity parameters were assessed with different methods between studies, used alone or in combination. While the most frequent metrics for evaluating α-diversity were the Observed species and the Shannon and Chao1 indexes, the unweighted and/or weighted UniFrac distances and Bray–Curtis dissimilarity were used to evaluate β-diversity (Table 3 and Table 4). In the 12 studies comparing the mucosal microbiome between CRC patients and healthy controls, five assessed α-diversity [35,36,58,60,69], with inconsistent results: one study reporting significantly higher α-diversity in CRC patients [35], one showing significantly lower α-diversity in CRC [69], and the remaining three reporting no statistically significant differences [36,60] or showing inconsistencies between the text and the presented figures [58]. Eight studies assessed β-diversity [34,35,36,57,60,65,68,69], and four of them showed that the structure of the mucosal microbiome of CRC patients was significantly different from that of healthy controls [35,57,60,65] (Table 3).

In the 33 studies comparing the microbiota of tumor and non-cancerous tissues in CRC patients, 22 evaluated α-diversity, with 12 showing no statistically significant differences between tumor and normal mucosa [37,42,44,45,47,48,53,59,60,63,64,65], eight reporting significantly lower microbial diversity in cancer tissues [40,46,50,51,55,62,66,70], and one reporting significantly higher diversity in cancer tissues [39] (Table 4). One study stated lower α-diversity in CRC, but without statistical support [52]. Three studies indicated different α-diversity results when using different indexes [40,55,66]. Twenty studies assessed β-diversity, with five showing that the structure of the microbial communities could distinguish cancer from non-cancerous tissues [19,50,54,62,64], seven showing no statistically significant differences [37,40,48,55,57,60,63], and the remaining eight studies reporting differences or similarities in the microbiota structure, but without statistical methods supporting the findings [42,44,45,46,49,52,53,70]. The use of different metrics and statistical tests to determine microbial diversity is a limitation in the comparability of the results reported.

### 3.4. Microbial Taxonomy Findings

The reported taxonomic levels varied throughout different studies, with predominant analysis and detection of phyla and genera. Despite some studies identifying taxa to the species level, it was necessary to approach these results with caution considering the intrinsic limitation of 16S rRNA short-amplicon sequencing in providing reliable detection at the lower taxonomic level of species.

While some results on bacterial taxa were consistent between studies, others were only significant in one study, or differed in the direction of the associations. For example, the phylum Firmicutes was found to be significantly enriched in the normal mucosa in four studies [39,41,44,62] and significantly enriched in CRC tissues in one study [58]. Various studies did not present overall taxonomic comparisons between CRC and the non-cancerous mucosa or the respective statistical analysis [19,34,38,47,49,69]. Three studies did not identify significant differences in the overall microbiota composition in CRC tissues and the paired non-cancerous mucosal tissues [51,55,57]. A detailed overview of the statistically significant taxonomic findings in the different studies can be found in Supplementary Table S5.

Table 5 presents the qualitative synthesis of the microbial taxonomic relationships with CRC, considering strong positive and negative associations. Strong positive associations were identified for 12 taxa. A strong positive association was identified for the phylum Fusobacteria, which was enriched in the CRC mucosa in comparison with both the paired non-cancerous mucosa [37,39,41,44,48,62,66,70] and the mucosa of healthy controls [58,60,65,68]. Strong positive associations were likewise identified for the family *Fusobacteriaceae* and for the genus *Fusobacterium*, the latter having the highest number of studies reporting statistically significant associations. A significant enrichment of *Fusobacterium* was reported in the mucosa of CRC patients in six studies that compared it with the mucosa of healthy controls [35,57,58,59,61,68], and in 19 studies that compared it with the non-cancerous tissue [37,39,42,44,46,47,48,52,53,54,58,59,61,62,63,64,66,67,70]. A strong positive association was identified for *Fusobacterium nucleatum*, with three studies reporting its enrichment in the CRC mucosa in comparison with the non-cancerous mucosa [37,43,56]. Additional strong positive associations were identified for *Campylobacter* [37,42,44,48,57,59,61,62,64,67,68,70], *Parvimonas* [37,42,44,57,59,61,63,67,68,70], *Peptostreptococcus* [42,44,54,57,58,59,61,63,67,68], *Streptococcus* [33,37,48,53,57,58,60,61,67], and *Granulicatella* [37,44,57,59,61]. All of these genera were found to be significantly enriched in CRC in comparisons with both healthy controls and with the non-cancerous mucosal tissue. Strong positive associations with CRC were also identified for *Selenomonas* [44,62,67,70] and *Gemella* [37,44,59] in studies that compared CRC tissue with the non-cancerous tissue. At the species level, *Bacteroides fragilis* had a strong positive association with CRC both in comparison with the non-cancerous tissue [43,52,59] and with healthy controls [35,59,65,68]. Overall, six genera—*Fusobacterium*, *Campylobacter*, *Parvimonas*, *Peptostreptococcus*, *Streptococcus*, and *Granulicatella*—were identified as strongly associated and enriched in CRC tissues in both types of studies (Table 5). These genera were identified in patients from both Eastern and Western origins (Table 5).

Taxa with suggestive positive associations with CRC are shown in Supplementary Table S6, and include, among others, the orders Campylobacteriales, Fusobacteriales [62,70], and Clostridiales [35,57], the families *Campylobacteriaceae* [62,70], *Gemellaceae* [67,70], and *Streptococcaceae* [42,60], the genera *Escherichia-Shigella* [57,58], *Oscillospira* [35,59], and *Porphyromonas* [33,57], and the species *Gemella morbillorum* [43,52] and *Parvimonas micra* [43,52].

Strong negative associations were identified for 18 taxa. A strong negative association was identified for the family *Ruminococcaceae,* which was reported as enriched in the non-cancerous mucosa of CRC patients in seven studies [39,40,53,60,62,67,70]. Likewise, there was a strong negative association between CRC and the genus *Ruminococcus* [37,42,53,62,66,67]. Further strong negative associations were identified for the genera *Parabacteroides* [37,40,42,44,46,53,60,62,70], *Faecalibacterium* [37,40,53,60,62], *Pseudomonas* [42,44,50,58], *Acinetobacter* [44,52,70], *Akkermansia* [53,64,67], *Bacillus* [40,44,61], *Bifidobacterium* [50,62,70], *Collinsella* [50,62,67], and the species *Faecalibacterium prausnitzii* [39,46,60], all in studies that compared CRC tissue with the non-cancerous mucosal tissue. *Klebsiella* [35,36,57] and *Propionibacterium* [35,58,68] had strong negative associations with CRC in studies comparing the microbiota of CRC patients with that of healthy controls. *Blautia* had a strong negative CRC association in both types of comparisons [37,46,57,59,60,68].

Taxa with suggestive negative associations with CRC included the family *Bifidobacteriaceae* [62,70] and the genera *Methylobacterium* [40,61], *Oscillibacter* [50,52], *Paraprevotella* [40,42], and *Veillonella* [54,67], all reported in studies comparing tumor and non-cancerous mucosal tissues. Additional suggestive negative associations with CRC were identified for the genera *Acinetobacter*, *Brevundimonas*, *Faecallibacterium*, *Neisseria*, *Pedobacter*, and *Stenotrophomonas* in comparisons of CRC patients with healthy controls. The full set of taxa and the respective associations are detailed in Supplementary Table S6.

Finally, microbiome correlation network analysis was performed in six of the included studies [33,39,50,59,69,70], most of them reporting positive and/or negative correlations between *Fusobacterium* and other taxa, and in general, bacteria found enriched in CRC tissues tended to co-occur.

### 3.5. Microbiota and Clinicopathological Features

Although the relationships between the microbiota and CRC clinical and pathological parameters were not the primary outcomes of this review, we summarized the major findings of the 14 included studies that performed these analyses [34,35,41,44,46,47,48,51,52,53,57,58,59,60]. One study reported differences in the microbiota of late-stage CRC, with decreased α-diversity, increased abundance of *Fusobacterium*, *Peptostreptococcus*, and *Streptococcus*, and lower levels of *Akkermansia*, *Ruminococcus*, *Granulicatella*, *Lactobacillus*, and, *Bacteroides fragilis* [51]. An enrichment of *Fusobacterium* and *Campylobacter* in the tumor in comparison to the normal mucosa was also reported in patients with T4 tumors [48]. Other studies, however, reported *Fusobacterium* as more abundant in early-stage CRC [46,59]. Enrichment of *Parvimonas*, *Gemella*, and *Leptotrichia* [59], *Eikenella corrodens* and *Eubacterium ventriosum* [68], and *Bacteroides fragilis* [46] was also reported in early- compared to late-stage CRC. Low levels of *Bacteroides*, *Blautia*, *F. prausnitzii*, *Sutterella*, *Collinsella aerofaciens* and *Alistipes putredinis* in early-stage CRC were also reported [59], as well as increased abundance of *Prevotella intermedia*, *Harryflintia acetispora* and *Dialister pneumosintes* in advanced CRC [68]. Thomas et al. found an increased abundance of *Coprococcus*, *Dorea*, *Roseburia*, and *Mogibacterium* in CRC with lymph node metastasis [35]. Mira-Pascual et al. analyzed the relationship between tumor stage and the microbiota composition, and identified lower abundance of *Staphylococcus* in T2 vs. T3 tumors, and within the T3 stage, *Streptococcus* was significantly more abundant in tumor than in healthy tissues [34]. Kinross et al. identified a cluster of bacteria, comprising *Lachnospiracea intertie sedis*, *Streptococcus, Prevotella*, and *Paraprevotella*, associated with patients with T4 adenocarcinomas, and poor tumor differentiation [47]. Poorly differentiated tumors had overrepresentation of *Fusobacterium*, *Streptococcus*, *Solobacterium*, and *Clostridium XI*, and underrepresentation of *Subdoligranulum*. This study also reported an association between increased *Bacteroides* with extramural vascular invasion and lower levels of *Roseburia* with the presence of lymphovascular invasion. One study evaluated the microbiota according to the histological subtype of CRC and identified increased *Fusobacterium* and *Campylobacter* and low levels of *Brevundimonas* in adenocarcinoma vs. normal tissue, whereas no such differences were identified in the mucinous subtype [48]. The same study also reported higher relative abundance of *Fusobacterium* and *Campylobacter*, and decreased *Brevundimonas* in CRC tissue compared to normal mucosa in patients with lower survival time (below 20 months), whereas no such differences could be identified in patients with higher survival (20–40 months and over 40 months) [48].

Six studies evaluated the relationship between the colonic microbiota and tumor location in CRC, with differences in the microbial composition between proximal and distal cancer [52,53,57,58,63,65]. *Halomonas* and *Shewanella* [57], *Fusobacterium*, *Escherichia-Shigella*, and *Leptotrichia* [58] and *Parvimonas micra* [52] were predominant in distal CRC, whereas *Faecalibacterium*, *Blautia*, and *Clostridium* [57], *Prevotella*, *Pyramidobacterium*, *Selenomonas*, and *Peptostreptoccus* [58] prevailed in proximal CRC. In contrast, in another study, *Fusobacterium* and *Bacteroides fragilis* were found more abundant in proximal tumors [52]. Others reported *Akkermansia muciniphila*, *Granulicatella adiacens*, *Streptococcus intermedius,* and *Gemella haemolysans* as significantly more abundant, and *Alistipes* spp., *Bacteroides* spp., and *Parabacteroides distasonis* as significantly less abundant, in the sigma-descending colon than in the ascending colon [65]. One study reported an increase in microbial richness from proximal colon to rectal cancer [58], while these differences were not found in samples of healthy controls [57]. Overall, no major consensus could be identified as to which specific taxon is associated with different clinicopathological features.

## 4. Discussion

The characterization of the microbiome–host interactions in CRC is crucial for generating knowledge that bridges the gap towards the understanding of the mechanisms of colorectal carcinogenesis mediated by microorganisms. For that, it is key to have solid information on the microbiota that is present in the tissues and likely plays a more important role in promoting chronic inflammation and tumorigenesis in CRC [25], rather than the more variable and non-adhered fecal microbiota [23,24]. Such knowledge may ultimately be used in novel strategies that aim to prevent, detect, and treat CRC.

Systematic reviews presenting evidence of gut microbiota differences between CRC and healthy status, based on the fecal microbiota [26] or on the combination of fecal and tissue microbiota [32], already exist. As per our knowledge, this is the first systematic review focusing entirely on the microbiota in tissue samples in the context of CRC. This systematic review included 39 studies that examined the differences between the mucosal microbiota in patients with CRC and healthy controls, and within CRC patients, the differences between the microbiota in the cancerous and in the non-cancerous tissues. We consider that these results reflect the best available evidence about microbiota composition and colorectal health. Although a meta-analysis was not performed due to the considerable heterogeneity in the parameters evaluated by the different studies, a qualitative synthesis of microbial taxonomy was presented.

The large variability in findings for both α- and β-diversity across studies did not allow drawing major conclusions. Reduced microbial diversity appears to be a key aspect of many disorders [71], and significantly lower diversity in cancer tissues was reported in nine studies [40,46,50,51,55,62,66,69,70]. Still, a large number of studies reported no significant differences [36,37,42,44,45,47,48,53,59,60,63,64,65]. In fact, significantly lower diversity of the microbiota in fecal samples from patients with CRC compared with normal subjects has been described [72,73,74]. As for the β-diversity, the microbial structure could distinguish CRC patients from healthy controls in 50% of the studies. In contrast, in 75% of the studies comparing cancerous with non-cancerous tissues from the same patient, the microbiota could not distinguish the two conditions. This suggests that the mucosal microbiota may have an influence not only in the tumor tissue but also in the surrounding non-cancerous tissues.

The microbial taxonomic findings showed that the mucosal microbiota differs not only between patients with CRC and healthy individuals, but also in CRC patients between their cancerous and non-cancerous tissues. Several taxa were found consistently associated with CRC across different studies. We defined a core of six genera enriched in CRC, including *Fusobacterium*, *Campylobacter*, *Parvimonas*, *Peptostreptococcus*, *Streptococcus*, and *Granulicatella*. Importantly, these genera were identified in studies including patients from Eastern and Western origins, reinforcing their positive association with CRC, independently of the patient origin. These bacteria tended to co-occur, clustering into groups with positive correlations with each other and negative correlations with networks of bacteria depleted in CRC [59].

High levels of *F. nucleatum* and *Campylobacter* have been associated with poor outcomes of CRC, with bacterial load increasing with disease progression [48,75,76]. Importantly, *F. nucleatum* has been found to promote CRC resistance to chemotherapy, being abundant in tumor tissues of patients with recurrence after chemotherapy, and by targeting innate immune signaling, specific microRNAs, and autophagy [77]. This suggests that decreasing the abundance of *Fusobacterium* may be helpful to reduce not only CRC progression but also resistance to chemotherapy.

*Fusobacterium*, and in particular *F. nucleatum*, attaches to and invades human cells via the FadA adhesin, activating β-catenin signaling, increasing expression of transcription factors LEF/TCF and NF-κB and cytokines IL-6, IL-8, and IL-18, thus being able to generate a pro-inflammatory microenvironment and promote growth of CRC cells [78]. Mechanistic evidence in CRC pathogenesis has also been identified for *Campylobacter* and *Streptococcus*. *C. jejuni* can promote CRC tumorigenesis through cytolethal distending toxin in germ-free ApcMin/+ mice [79]. *Streptococcus gallolyticus* can selectively colonize tumor cells and promote chronic inflammation and angiogenesis, contributing to the maintenance and development of pre-existing neoplastic lesions [80,81]. Interestingly, *Fusobacterium* has a strong ability to induce co-aggregation, and it has been suggested that it facilitates the internalization of normally non-invasive bacteria into epithelial cells, including *Campylobacter* spp. and *Streptococcus* spp. [82,83].

Additional mechanistic data for a role of other bacterial taxa in CRC pathogenesis have been increasing. *Parvimonas micra* was shown to promote intestinal carcinogenesis in ApcMin/+ and germ-free mice, through alteration of immune responses, increased expression of pro-inflammatory cytokines including TNF-α, IL-6 and IL-12, and proliferation of colon cells [84]. *Peptostreptococcus anaerobius* surface protein PCWBR2 interacts with host cell α2/β1 integrins, enhancing cell proliferation and a pro-inflammatory immune environment, which promotes CRC development in mice [85]. Furthermore, *P. anaerobius* interacts with TLR2 and TLR4 to increase intracellular levels of reactive oxidative species leading to cell proliferation, intestinal dysplasia, and CRC progression [86]. Enterotoxigenic *Bacteroides fragilis* has been also associated with colorectal carcinogenesis, through the fragilysin virulence metalloprotease that increases epithelial cell permeability, inflammation, and TCF-dependent β-catenin nuclear signaling [87,88].

In this systematic review, other bacterial taxa were defined to have strong negative associations with CRC, including *Faecalibacterium*, *Parabacteroides*, and *Blautia*. *Faecalibacterium*, namely *F. prausnitzii*, was found to be depleted in cancer tissues in comparison with the non-cancerous mucosa of CRC patients, suggesting that the microenvironment at the cancer site is not favorable to this bacterium. *F. prausnitzii* is the major butyrate producer in the colon, inhibiting NF-κB and promoting IL-10 secretion, leading to inhibition of the production of inflammatory mediators [89]. *F. prausnitzii* and its metabolites were shown to ameliorate colitis lesions [90]. *Parabacteroides distasonis* suppresses colonic tumorigenesis and maintains the intestinal epithelial barrier in azoxymethane-treated mice [91]. *Blautia* produces bacteriocins that maintain microbiome homeostasis and prevent inflammation by upregulation of intestinal regulatory T cells [92].

CRC carcinogenesis is a long process that may take decades to develop [93]. Dysbiotic changes in the gut bacterial community associated with inflammation and tumorigenesis were shown to occur prior to the first signs of macroscopic tumor formation [94]. Still, it is difficult to ascertain the true meaning of the associations between the microbial taxa and CRC. Changes that occur in the tumor microenvironment along with disease progression may lead to selective pressure on the microbial community [95]. As a result, there may be a temporally different CRC-associated microbiota during tumor development, and bacteria with pro-carcinogenic features that initiate CRC carcinogenesis, may be outcompeted by opportunistic bacteria that thrive and proliferate in the tumor microenvironment [95].

Adding to the above and despite having defined bacterial genera with strong CRC associations, many taxa inconsistencies between studies were also identified. Apart from methodologic differences, one should also take into consideration the microbiome functional redundancy, where different taxa may have similar metabolic signatures and functions [96]. Thus, the lack of consistency found for some bacterial taxa may still be in line with consistency in functionality.

Although the reviewed evidence revealed differences between the microbial communities of CRC and non-cancerous tissues (from both healthy controls and CRC patients), the capacity to draw definite conclusions was limited by different aspects. The methodologies used for DNA extraction, sample handling, and PCR were heterogeneous between studies. Adding to this, the absence of negative controls throughout sample processing and the use of dissimilar sequencing technologies may have also contributed to variation in the results [97]. Another important issue was the selection of the hypervariable regions of the 16S rRNA gene analyzed, as the lack of standardized usage of variable regions can lead to distinct taxonomic profiles and diversity estimates [98]. Finally, differences in bioinformatics and statistical analyses also contributed to inconsistencies between studies. For example, taxonomic classification was performed using distinct taxonomic classifiers and reference databases, and different versions of both. This, coupled with the application of methods with different assumptions and statistical power, can also lead to heterogeneity in results. Other limiting factors were the small number of subjects included and their poor characterization, with minimal reporting of factors that interfere with the microbiome, such as the use of antimicrobial therapy, probiotics, and body mass index. In addition, bowel cleansing for colonoscopy, used as a selection method in some studies, can influence the colon microbiome and was not considered as an exclusion criterion. Considering the importance of these factors as potential confounders, we included in the study quality assessment the use of antibiotics as an exclusion criterion. While studies comparing tumor and matched non-cancerous mucosa do not present variation in age, sex, diet, and other characteristics, those comparing CRC and healthy controls had differences in these potential confounders, which may have influenced the results. Another limiting factor was variation in populations from distinct geographical regions, with different genetic backgrounds and diet and lifestyle habits that may have impacted colorectal microbiome composition [99]. This is in line with the findings from Allali et al. reporting significantly different microbial diversity between US and Spanish cohorts [37]. Finally, none of the included studies had longitudinal follow-up, thus not allowing the establishment of causal relationships between the microbiota and the maintenance of health or the development of disease.

## 5. Conclusions

Although having herein defined a core microbiota associated with CRC, many microbiota features were inconsistent and lacked strong evidence to draw definite conclusions about their role in CRC. It is, therefore, urgent to standardize methodologies for microbiome analysis and reporting in order to increase the comparability of results. Future, well-designed prospective studies including large numbers of subjects and taking into consideration potential confounding factors will be key to clarifying the causal association between the microbiome and CRC. Ultimately, a better understanding of the CRC microbiome and its interaction with the host will contribute to novel microbiome-based prevention, diagnosis, and treatment strategies aimed at controlling and decreasing the CRC burden.

## Figures and Tables

**Table 1:** Baseline characteristics of studies determining the mucosal colorectal microbiota of CRC patients and healthy controls.

First Author, Year	Country	No. Participants	Study Participants (Males) Age Mean or Median ± SD (Range)		Recruitment	Exclusions
			CRC	Healthy Controls		
Geng, 2014 [33]	China	18	8 (4) Mean 56.9 ± 14.4	10 (NR) NR	CRC: Undergoing colonoscopy HC: volunteers	NR
Gao, 2015 [58]	China	61	31 (15) Mean 67 ± 7.2	30 (14) Mean 70 ± 5.1	CRC: Undergoing CRC surgery HC: Undergoing colonoscopy	HC: BMI > 30 kg/m^2^; HC and CRC: use of antibiotics within 2 months, regular use of NSAIDs, statins, or probiotics; chronic bowel disorders, food allergies/dietary restrictions; pre-operative radiation or chemotherapy
Mira-Pascual, 2015 [34]	Spain	12	7 (7) Mean 71.1 ± 10.1	5 (3) Mean 58.8 ± 10	Undergoing CRC screening	NR
Nakatsu, 2015 [59]	China	DC: 113 VC: 75	DC: 52 (31) Mean 67.85 ± 13.18 VC: 50 (26) Mean 61.34 ± 9.97	DC: 61 (25) Mean 60.813 ± 5.99 VC: 25 (10) Mean 41.28 ± 7.87	Undergoing CRC screening	Personal history of CRC, IBD, prosthetic heart valve or vascular graft surgery; contraindications for colonoscopy
Thomas, 2016 [35]	Brazil	36	18 (10) Mean 59.3 ± 8.8	18 (9) Mean 55.2 ±15.7	HC: Undergoing exploratory colonoscopy CRC: Undergoing CRC surgery	HC and CRC: use of antibiotics 4 weeks before sample collection; CRC: neoadjuvant therapy prior to tissue collection; IBD, hereditary cancer syndromes
Flemer, 2017 [57]	Ireland	115	59 (37) Range 41–90	56 (24) Range 27–29	HC: Undergoing s colonoscopy CRC: Undergoing CRC surgery	HC and CRC: Personal history of CRC, IBD, or IBS; CRC: use of antibiotics the month prior to surgery
Richard, 2018 [60]	Italy	27	CAC: 7(5) Mean 50.7 ± 10 SC: 10 (5) Mean 68.8 ± 12.1	10 (7) Mean 48.3 ± 13.4	HC: Undergoing routine screening CRC: Undergoing CRC surgery	HC: History/clinical symptoms of intestinal disorders and endoscopic/histological signs of cancer or IBD; HC and CRC: Infectious colitis, coagulation disorders, anticoagulant therapy; use of antibiotics/antifungal therapy 2 months before inclusion
Zhang, 2019 [36]	China	23	9 (6) Mean 62.6 ± 8.9	14 (7) Mean 44.1 ± 15	Undergoing CRC screening	IBS; use of antibiotics or probiotics 30 days or infectious gastroenteritis 60 days before colonoscopy
Wang Y, 2020 [61]	China	101	75 (48) Mean 63.4 (Range 29–82)	26 (17) Mean 51.7 (Range 21–71)	HC: Undergoing colonoscopy CRC: Undergoing CRC surgery	NR
Nardelli, 2021 [65]	Italy	40	20 (10) Mean 69.4	20 (10) Mean 53.2	HC: Undergoing colonoscopy CRC: Undergoing CRC surgery	IBD or IBS; use of antibiotics, pro/prebiotics, antiviral, or corticosteroids 2 months prior to sample collection
Osman, 2021 [68]	Malaysia	36	18 (12) Mean 64.88 ± 2.34	18 (11) Mean 54.44 ± 2.91	Undergoing colonoscopy and tumor removal surgery	History of cancer, IBD and polyps; use of antibiotics 3 months prior to radiotherapy or chemotherapy prior to surgery
Wang, 2021 [69]	China	60	30 (17) Mean 63.9 ± 6.58	30 (15) Mean 52.17 ±9.02	HC: Undergoing colonoscopy CRC: Undergoing surgery	History of cancer, Peutz–Jeghers or Lynch syndromes; use of antibiotics/NSAIDS 1 month prior to sample collection

Abbreviations: CAC: colitis-associated colorectal cancer; CRC: colorectal cancer; DC: discovery cohort; HC: healthy controls; IBD: inflammatory bowel disease; IBS: irritable bowel syndrome; NR: not reported; NSAIDs: non-steroidal anti-inflammatory drugs; SC: sporadic colorectal cancer; SD: standard deviation; VC: validation cohort.

**Table 2:** Baseline characteristics of studies determining the microbiota of CRC and non-cancerous mucosal tissues.

First Author, Year	Country	No. Participants (Males)	Age Mean ± SD Median (Range)	Recruitment	Exclusions	NCT Distance from Tumor
Marchesi, 2011 [19]	The Netherlands	6 (5)	Mean 63.5 (49–71)	Undergoing CRC surgery	NR	5–10 cm
Chen, 2012 [40]	China	46 (NR) For analysis: 27 (14)	Mean 61 (37–81)	Undergoing CRC surgery	Diabetes, infectious diseases, particular diets; use of antibiotics within 1 month of sample collection	Pa2t: 2–5 cm; Pa10t: 10–20 cm
Geng, 2013 [45]	China	8 (4)	Mean 56.9 ± 14.4	Undergoing CRC screening	NR	NR
Zeller, 2014 [56]	Germany	38 (25)	Mean 61.7 ± 13.5 (34–90)	Undergoing CRC surgery	Previous colon or rectal surgery, CRC, inflammatory or infectious injuries of the intestine; need for emergency colonoscopy	NR
Allali, 2015 [37]	USA Spain	USA: 22 (11) Spain: 23 (15)	Mean 63.6 (42–88) Mean 69.8 (49–85)	Tissue bank Undergoing CRC surgery	NR	USA: NR Spain: 5 cm
Burns, 2015 [39]	USA	44 (12)	Mean 64.9 ± 16.7 (17–91)	Biobank	NR	NR
Gao, 2015 [58]	China	31 (15) For analysis: 20 NCT	Mean 67 ± 7.2	Undergoing CRC surgery	Use of antibiotics within 2 months, regular use of NSAIDs, statins, or probiotics; chronic bowel disorders, food allergies/dietary restrictions; pre-operative radiation or chemotherapy	5 cm
Nakatsu, 2015 [59]	China	DC: 52 (31) VC: 50 (26)	DC: Mean 67.85 ± 13.18 VC: Mean 61.34 ± 9.97	Undergoing CRC screening	Personal history of CRC, IBD, prosthetic heart valve or vascular graft surgery; contraindications for colonoscopy	≥4 cm
Brim, 2017 [38]	USA	10 (5)	Range 41–88	Undergoing CRC surgery	NR	NR
Drewes, 2017 [43]	Malaysia	23 (12)	Mean 62.22 ± 11.99	Undergoing CRC surgery	Personal history of CRC or IBD; pre-operative radiation or chemotherapy	NR (as far as possible)
Flemer, 2017 [57]	Ireland	59 (37)	Range 41–90	Undergoing CRC surgery	Personal history of CRC, IBD, or IBS; use of antibiotics the month prior to surgery	OFFD and OFFP: 2–5 cm; UDD and UDP: 10–30 cm from the tumor
Gao, 2017 [44]	China	65 (35)	Mean 63.49 ± 1.46	Undergoing CRC surgery	Use of antibiotics or probiotics within 4 weeks, acute diarrhea, adenoma or polyps, IBD, IBS	>5 cm
Kinross, 2017 [47]	UK	18 (10)	Median 76 (55–85)	Undergoing CRC surgery	Previous colorectal surgery, undergoing emergency surgery; pre-operative chemotherapy or radiotherapy; use of antibiotics or probiotics 6 weeks prior to surgery; history of FAP or IBD	5 cm and 10 cm
Cremonesi, 2018 [41]	Germany	31 (21) For analysis: 27	67.5 (35–82)	Undergoing CRC surgery	NR	NR
Hale, 2018 [46]	USA	106 (57)	Mean 65.3 (23–90)	Undergoing CRC surgery	Radio or chemotherapy 2 weeks before enrollment	NR (adjacent and distal)
Loke, 2018 [50]	Malaysia	17 (7)	Mean 62.47 (41–84)	Undergoing CRC surgery	Pre-operative radiation or chemotherapy; history of CRC or IBD	NR
Richard, 2018 [60]	Italy	CAC: 7(5) SC: 10 (5)	CAC: Mean 50.7 ± 10 SC: Mean 68.8 ± 12.1	Undergoing CRC surgery	Infectious colitis, coagulation disorders, anti-coagulant therapy; use of antibiotics or antifungal therapy 2 months before inclusion	<5 cm
de Carvalho, 2019 [42]	Brazil	152 (81) For analysis: 15	Mean 60.63 ± 13.7	Undergoing CRC surgery	NR	NR
Leung, 2019 [48]	Australia	19 (9)	Mean 64.7 ± 15.4	Undergoing CRC surgery	NR	Proximal resection margin
Liu, 2019 [49]	China	8 (5)	Mean 61.3 ± 10.1 (50–78)	Undergoing CRC surgery	NR	2 cm
Saffarian, 2019 [52]	France	58 (37)	Mean 68.98 (23–92)	Undergoing CRC surgery	Undergoing chemotherapy, radiotherapy, or antibiotic treatment	15–20 cm
Pan, 2020 [51]	China	23 (11)	Range: 49–70	Undergoing CRC surgery	Use of antibiotics prior to sample collection	>5 cm
Sheng, 2020 [53]	China	66 (38)	Range: 35–94	NR	Radiotherapy or chemotherapy before surgery; use of antibiotics, NSAIDs, statins, or probiotics 3 months before surgery; family history of CRC; IBD; diabetes; hypertension; food allergies	>10 cm
Wang Q, 2020 [54]	China	36 (NR)	NR	Undergoing CRC surgery	Use of antibiotics or probiotics 4 weeks before surgery; undergoing radiotherapy or chemotherapy; diabetes; infectious diseases	>5 cm
Wang Y, 2020 [61]	China	75 (48)	Mean 63.4 (29–82)	Undergoing CRC surgery	NR	Adjacent and off tumor
Wirth, 2020 [55]	Germany	6 (NR)	NR	Undergoing CRC surgery	NR	NR
Choi, 2021 [62]	Republic of Korea	51 (51)	Range: 43–86	Undergoing CRC surgery	NR	NR
Liu, 2021 [63]	China	DC: 11 (8) VC: 10 (8)	DC: Mean 64.91 ± 15.20 VC: Mean 65.33 ± 7.54	NR	NR	NR
Malik, 2021 [64]	USA	51 (30)	62 ± IQR 20	Undergoing CRC surgery	Hereditary CRC syndromes, IBD; neoadjuvant treatment	NR
Nardelli, 2021 [65]	Italy	20 (10)	Mean 69.4	Undergoing CRC surgery	IBD or IBS; use of antibiotics, pro/prebiotics, antivirals, or corticosteroids 2 months prior to sample collection	NR
Niccolai, 2021 [66]	Italy	45 (NR)	Range: 30–90	Undergoing CRC surgery	Previous cancer surgery, chemo or radiotherapy; use of immunosuppressives, antibiotics, or probiotics in the previous 2 months; cancer, IBD	NR
Okuda, 2021 [67]	Japan	29 (15)	Range 37–94	Underwent CRC surgery	CRC with FAP; IBD	3 cm
Zhang, 2021 [70]	China	136 (81) For analysis 101 (58)	Median 64 (21–88) For analysis: Median 64 (21–88)	Undergoing CRC surgery	No chemo or radiotherapy and no antibiotics 1 month before resection	NR (as far as possible)

Abbreviations: AM: adjacent mucosa; CAC: colitis-associated colorectal cancer; CRC: colorectal cancer; DC: discovery cohort; FAP: familial adenomatous polyposis; IBD: inflammatory bowel disease; IBS: irritable bowel syndrome; NCT: non-cancerous tissue; NR: not reported; NSAIDs: non-steroidal anti-inflammatory drugs; OFFD: off-distal; OFFP: off-proximal; Pa2t: matched paracancerous tissue 2–5 cm; Pa10t: matched paracancerous tissue 10–20 cm; SC: sporadic colorectal cancer; SD: standard deviation; UDD: undiseased distal; UDP: undiseased proximal; VC: validation cohort.

**Table 3:** Summary of the diversity findings in the colorectal microbiota of CRC patients and healthy controls.

	α-Diversity		β-Diversity	
First Author, Year	Measure	Findings in CRC	Measure	Findings
Geng, 2014 [33]	NR	NR	NR	NR
Gao, 2015 [58]	Shannon, Simpson, Chao1 and ACE indexes	Inconsistent between text description and figures	NR	NR
Mira-Pascual, 2015 [34]	NR	NR	UniFrac	Distinguished CRC from HC ^‡^
Nakatsu, 2015 [59]	NR	NR	NR	NR
Thomas, 2016 [35]	Observed species, Shannon and Simpson indexes	Significantly higher	Unweighted and weighted UniFrac; Bray–Curtis dissimilarity	Distinguished CRC from HC
Flemer, 2017 [57]	NR	NR	Unweighted and weighted UniFrac; Spearman rank distance	Distinguished CRC from HC
Richard, 2018 [60]	Chao1 index Observed species and Shannon index	NS Significantly lower in CAC	Bray–Curtis dissimilarity	Distinguished HC from SC and CAC Distinguished SC from CAC
Zhang, 2019 [36]	Shannon and Chao1 indexes	NS	Unweighted UniFrac	Similar between CRC and HC ^‡^
Wang Y, 2020 [61]	NR	NR	NR	NR
Nardelli, 2021 [65]	NR	NR	Weighted UniFrac	Distinguished CRC from HC
Osman, 2021 [68]	NR	NR	Unweighted UniFrac	Distinguished CRC from HC ^‡^
Wang, 2021 [69]	Observed species, Shannon, Chao, and ACE indexes	Significantly lower	Weighted UniFrac	Distinguished CRC from HC ^‡^

Abbreviations: CAC: colitis-associated colorectal cancer; CRC: colorectal cancer; HC: healthy controls; NR: not reported; NS: no statistically significant differences; SC: sporadic colorectal cancer; ^‡^ without statistical analysis.

**Table 4:** Summary of the diversity findings in the mucosal microbiota of CRC and non-cancerous mucosal tissues.

	α-Diversity		β-Diversity	
First Author, Year	Measure	Findings in CRC	Measure	Finding
Marchesi, 2011 [19]	NR	NR	Libshuff analysis	Distinguished CRC from NCT
Chen, 2012 [40]	Shannon index Chao index	Significantly lower NS	Unweighted UniFrac	NS
Geng, 2013 [45]	Observed species	NS	UniFrac	Distinguished CRC from NCT ^‡^
Zeller, 2014 [56]	NR	NR	NR	NR
Allali, 2015 [37]	Phylogenetic diversity and observed species	NS	Unweighted UniFrac	NS
Burns, 2015 [39]	Phylogenetic diversity, Shannon and Inverse Simpson’s indexes	Significantly higher	NR	NR
Gao, 2015 [58]	NR	NR	NR	NR
Nakatsu, 2015 [59]	Inverse Simpson’s index	NS	NR	NR
Brim, 2017 [38]	NR	NR	NR	NR
Drewes, 2017 [43]	NR	NR	NR	NR
Flemer, 2017 [57]	NR	NR	Unweighted and weighted UniFrac; Spearman rank distance	NS
Gao, 2017 [44]	ACE, Chao1, Shannon, and Simpson indexes	NS	Bray–Curtis dissimilarity	Distinguished CRC from NCT ^‡^
Kinross, 2017 [47]	Shannon index	NS	Bray–Curtis dissimilarity	NR
Cremonesi, 2018 [41]	NR	NR	NR	NR
Hale, 2018 [46]	Shannon index	Significantly lower	Unweighted and weighted UniFrac	Similar between CRC and NCT ^‡^
Loke, 2018 [50]	Observed species and Shannon index	Significantly lower	Unweighted UniFrac	Distinguished CRC from NCT
Richard, 2018 [60]	Observed species, Chao1, and Shannon indexes	NS	Bray–Curtis dissimilarity	NS
de Carvalho, 2019 [42]	Observed species, Chao1, Shannon indexes and Phylogenetic diversity	NS	Unweighted UniFrac	Similar between CRC and NCT ^‡^
Leung, 2019 [48]	Observed species, Chao1, Shannon, and Simpson	NS	Weighted UniFrac	NS
Liu, 2019 [49]	OTU number, Chao1, ACE, Shannon, and Simpson	NR	Weighted UniFrac	Similar between CRC and NCT ^‡^
Saffarian, 2019 [52]	Chao1 index	Lower ^‡^	Unweighted UniFrac	Similar between CRC and NCT ^‡^
Pan, 2020 [51]	Shannon index	Significantly lower in stage III	NR	NR
Sheng, 2020 [53]	Observed species, Chao1, Shannon, and Simpson	NS	Bray–Curtis dissimilarity	Similar between CRC and NCT ^‡^
Wang Q, 2020 [54]	NR	NR	Unweighted UniFrac	Distinguished CRC from NCT
Wang Y, 2020 [61]	NR	NR	NR	NR
Wirth, 2020 [55]	Shannon and Simpson indexes Chao1 and ACE indexes	Significantly lower NS	Unweighted and weighted UniFrac	NS
Choi, 2021 [62]	Shannon index and observed species	Significantly lower	Bray–Curtis dissimilarity	Distinguished CRC from NCT
Liu, 2021 [63]	Chao1 and Shannon indexes	NS	Bray–Curtis dissimilarity	NS
Malik, 2021 [64]	Observed species, Shannon and Evenness indexes	NS	Morisita–Horn dissimilarity	Distinguished CRC from NCT
Nardelli, 2021 [65]	Shannon index	NS	NR	NR
Niccolai, 2021 [66]	Chao1 and breakaway species richness Shannon index and Evenness	Significantly lower NS	NR	NR
Okuda, 2021 [67]	NR	NR	NR	NR
Zhang, 2021 [70]	Pielou’s evenness, Phylogenetic diversity, ACE, Chao, Shannon, and Simpson indexes	Significantly lower	Unweighted UniFrac	Similar between CRC and NCT ^‡^

Abbreviations: CAC: colitis-associated colorectal cancer; CRC: colorectal cancer; NR: not reported; NS: no statistically significant differences; NCT: non-cancerous tissue; SC: sporadic colorectal cancer; ^‡^ without statistical analysis.

**Table 5:** Qualitative synthesis showing the strong microbial taxonomic associations with CRC and the geographic origin of the populations in the studies.

	Microbial Taxa	CRC vs. HC N (%) Studies *	Origin		CRC vs. NCT N (%) Studies **	Origin	
			E	W		E	W
Phylum	Actinobacteria				4 (14%)	3	1
	Bacteroidetes				5 (17%)	1	4
	Fusobacteria	4 (40%)	2	2	8 (28%)	3	5
Family	*Fusobacteriaceae*				3 (10%)	2	1
	*Porphyromonadaceae*				4 (14%)	2	2
	*Rikenellaceae*				4 (14%)	2	2
	*Ruminococcaceae*				7 (24%)	5	2
Genus	*Acinetobacter*				3 (10%)	2	1
	*Akkermansia*				3 (10%)	2	1
	*Bacillus*				3 (10%)	3	0
	*Bifidobacterium*				3 (10%)	3	0
	*Blautia*	3 (30%)	2	1	4 (14%)	1	3
	***Campylobacter***	4 (40%)	3	1	9 (31%)	5	4
	*Collinsella*				3 (10%)	3	0
	*Faecalibacterium*				5 (17%)	3	2
	***Fusobacterium***	6 (60%)	4	2	19 (66%)	10	9
	*Gemella*				3 (10%)	2	1
	***Granulicatella***	3 (30%)	2	1	3 (10%)	2	1
	*Klebsiella*	3 (30%)	1	2			
	*Parabacteroides*				9 (31%)	5	4
	***Parvimonas***	4 (40%)	3	1	8 (28%)	6	2
	***Peptostreptococcus***	5 (50%)	4	1	6 (21%)	5	1
	*Propionibacterium*	3 (30%)	2	1			
	*Pseudomonas*				5 (17%)	4	1
	*Ruminococcus*				6 (21%)	3	3
	*Selenomonas*				4 (14%)	4	0
	***Streptococcus***	3 (30%)	2	1	7 (24%)	4	3
Species	*Bacteroides fragilis*	4 (40%)	2	2	3 (10%)	2	1
	*Faecalibacterium prausnitzii*				3 (10%)	0	3
	*Fusobacterium nucleatum*				3 (10%)	1	2

* [33,35,36,57,58,59,60,61,65,68]; ** [37,39,40,41,42,43,44,45,46,48,50,51,52,53,54,55,56,57,58,59,60,61,62,63,64,65,66,67,70]; E, Eastern origin; W, Western origin; Cells in magenta represent strong positive associations (≥3 studies in same direction; none in opposite direction); Cells in blue represent strong negative associations (≥3 studies in same direction; none in opposite direction).

## Supplementary Materials

The following supporting information can be downloaded at: https://www.mdpi.com/article/10.3390/cancers14143385/s1, Table S1: Microbiome methodological analysis of included studies of the mucosal microbiota of CRC patients and healthy controls; Table S2: Microbiome methodological analysis of included studies of CRC and non-cancerous mucosal tissues; Table S3: Quality of included CRC vs. HC studies based on the Newcastle–Ottawa quality assessment scale; Table S4: Quality of included CRC vs. NCT studies based on the Newcastle–Ottawa quality assessment scale; Table S5: Summary of the statistically significant microbial taxa identified in studies comparing CRC vs. HC and CRC vs. NCT; Table S6. Qualitative synthesis showing the strong and the suggestive microbial taxonomic associations with CRC. References [19,33,34,35,36,37,38,39,40,41,42,43,44,45,46,47,48,49,50,51,52,53,54,55,56,57,58,59,60,61,62,63,64,65,66,67,68,69,70] are cited in the supplementary materials.

## References

[1] Sung H., Ferlay J., Siegel R.L., Laversanne M., Soerjomataram I., Jemal A., Bray F. (2021). Global Cancer Statistics 2020: GLOBOCAN Estimates of Incidence and Mortality Worldwide for 36 Cancers in 185 Countries. CA Cancer J. Clin..

[2] Ferlay J., Ervik M., Lam F., Colombet M., Mery L., Piñeros M., Znaor A., Soerjomataram I., Bray F. Global Cancer Observatory: Cancer Today. https://gco.iarc.fr/today.

[3] Xie Y.H., Chen Y.X., Fang J.Y. (2020). Comprehensive review of targeted therapy for colorectal cancer. Signal Transduct. Target. Ther..

[4] Survival Rates for Colorectal Cancer. https://www.cancer.org/cancer/colon-rectal-cancer/detection-diagnosis-staging/survival-rates.html.

[5] Kuipers E.J., Grady W.M., Lieberman D., Seufferlein T., Sung J.J., Boelens P.G., van de Velde C.J., Watanabe T. (2015). Colorectal cancer. Nat. Rev. Dis. Primers.

[6] Müller M.F., Ibrahim A.E., Arends M.J. (2016). Molecular pathological classification of colorectal cancer. Virchows Arch..

[7] Fearon E.R. (2011). Molecular Genetics of Colorectal Cancer. Annu. Rev. Pathol. Mech. Dis..

[8] Hughes L.A.E., Simons C., van den Brandt P.A., van Engeland M., Weijenberg M.P. (2017). Lifestyle, Diet, and Colorectal Cancer Risk According to (Epi)genetic Instability: Current Evidence and Future Directions of Molecular Pathological Epidemiology. Curr. Colorectal Cancer Rep..

[9] Cho I., Blaser M.J. (2012). The human microbiome: At the interface of health and disease. Nat. Rev. Genet..

[10] Gilbert J.A., Blaser M.J., Caporaso J.G., Jansson J.K., Lynch S.V., Knight R. (2018). Current understanding of the human microbiome. Nat. Med..

[11] Allen J., Sears C.L. (2019). Impact of the gut microbiome on the genome and epigenome of colon epithelial cells: Contributions to colorectal cancer development. Genome Med..

[12] Ferreira R.M., Pereira-Marques J., Pinto-Ribeiro I., Costa J.L., Carneiro F., Machado J.C., Figueiredo C. (2018). Gastric microbial community profiling reveals a dysbiotic cancer-associated microbiota. Gut.

[13] Tilg H., Adolph T.E., Gerner R.R., Moschen A.R. (2018). The Intestinal Microbiota in Colorectal Cancer. Cancer Cell.

[14] Kostic A.D., Chun E., Robertson L., Glickman J.N., Gallini C.A., Michaud M., Clancy T.E., Chung D.C., Lochhead P., Hold G.L. (2013). *Fusobacterium nucleatum* potentiates intestinal tumorigenesis and modulates the tumor-immune microenvironment. Cell Host Microbe.

[15] Goodwin A.C., Destefano Shields C.E., Wu S., Huso D.L., Wu X., Murray-Stewart T.R., Hacker-Prietz A., Rabizadeh S., Woster P.M., Sears C.L. (2011). Polyamine catabolism contributes to enterotoxigenic *Bacteroides fragilis*-induced colon tumorigenesis. Proc. Natl. Acad. Sci. USA.

[16] Arthur J.C., Perez-Chanona E., Mühlbauer M., Tomkovich S., Uronis J.M., Fan T.J., Campbell B.J., Abujamel T., Dogan B., Rogers A.B. (2012). Intestinal inflammation targets cancer-inducing activity of the microbiota. Science.

[17] Castellarin M., Warren R.L., Freeman J.D., Dreolini L., Krzywinski M., Strauss J., Barnes R., Watson P., Allen-Vercoe E., Moore R.A. (2012). *Fusobacterium nucleatum* infection is prevalent in human colorectal carcinoma. Genome Res..

[18] Bullman S., Pedamallu C.S., Sicinska E., Clancy T.E., Zhang X., Cai D., Neuberg D., Huang K., Guevara F., Nelson T. (2017). Analysis of *Fusobacterium* persistence and antibiotic response in colorectal cancer. Science.

[19] Marchesi J.R., Dutilh B.E., Hall N., Peters W.H., Roelofs R., Boleij A., Tjalsma H. (2011). Towards the human colorectal cancer microbiome. PLoS ONE.

[20] Dai Z., Coker O.O., Nakatsu G., Wu W.K., Zhao L., Chen Z., Chan F.K., Kristiansen K., Sung J.J., Wong S.H. (2018). Multi-cohort analysis of colorectal cancer metagenome identified altered bacteria across populations and universal bacterial markers. Microbiome.

[21] Baxter N.T., Ruffin M.T.t., Rogers M.A., Schloss P.D. (2016). Microbiota-based model improves the sensitivity of fecal immunochemical test for detecting colonic lesions. Genome Med..

[22] Young C., Wood H.M., Fuentes Balaguer A., Bottomley D., Gallop N., Wilkinson L., Benton S.C., Brealey M., John C., Burtonwood C. (2021). Microbiome Analysis of More Than 2,000 NHS Bowel Cancer Screening Programme Samples Shows the Potential to Improve Screening Accuracy. Clin. Cancer Res..

[23] Parthasarathy G., Chen J., Chen X., Chia N., O’Connor H.M., Wolf P.G., Gaskins H.R., Bharucha A.E. (2016). Relationship Between Microbiota of the Colonic Mucosa vs Feces and Symptoms, Colonic Transit, and Methane Production in Female Patients With Chronic Constipation. Gastroenterology.

[24] Zoetendal E.G., von Wright A., Vilpponen-Salmela T., Ben-Amor K., Akkermans A.D., de Vos W.M. (2002). Mucosa-associated bacteria in the human gastrointestinal tract are uniformly distributed along the colon and differ from the community recovered from feces. Appl. Environ. Microbiol..

[25] Brennan C.A., Garrett W.S. (2016). Gut Microbiota, Inflammation, and Colorectal Cancer. Annu. Rev. Microbiol..

[26] Amitay E.L., Krilaviciute A., Brenner H. (2018). Systematic review: Gut microbiota in fecal samples and detection of colorectal neoplasms. Gut Microbes.

[27] Wirbel J., Pyl P.T., Kartal E., Zych K., Kashani A., Milanese A., Fleck J.S., Voigt A.Y., Palleja A., Ponnudurai R. (2019). Meta-analysis of fecal metagenomes reveals global microbial signatures that are specific for colorectal cancer. Nat. Med..

[28] Gethings-Behncke C., Coleman H.G., Jordao H.W.T., Longley D.B., Crawford N., Murray L.J., Kunzmann A.T. (2020). *Fusobacterium nucleatum* in the Colorectum and Its Association with Cancer Risk and Survival: A Systematic Review and Meta-analysis. Cancer Epidemiol. Biomark. Prev..

[29] Hussan H., Clinton S.K., Roberts K., Bailey M.T. (2017). Fusobacterium’s link to colorectal neoplasia sequenced: A systematic review and future insights. World J. Gastroenterol..

[30] Moher D., Liberati A., Tetzlaff J., Altman D.G., Group P. (2009). Preferred reporting items for systematic reviews and meta-analyses: The PRISMA statement. Ann. Intern. Med..

[31] Wells G.A., Shea B., O’Connell D., Peterson J., Welch V., Losos M., Tugwell P. The Newcastle-Ottawa Scale (NOS) for Assessing the Quality of Nonrandomised Studies in Meta-Analyses. http://www.ohri.ca//programs/clinical_epidemiology/oxford.asp.

[32] Huybrechts I., Zouiouich S., Loobuyck A., Vandenbulcke Z., Vogtmann E., Pisanu S., Iguacel I., Scalbert A., Indave I., Smelov V. (2020). The Human Microbiome in Relation to Cancer Risk: A Systematic Review of Epidemiologic Studies. Cancer Epidemiol. Biomark. Prev..

[33] Geng J., Song Q., Tang X., Liang X., Fan H., Peng H., Guo Q., Zhang Z. (2014). Co-occurrence of driver and passenger bacteria in human colorectal cancer. Gut Pathog..

[34] Mira-Pascual L., Cabrera-Rubio R., Ocon S., Costales P., Parra A., Suarez A., Moris F., Rodrigo L., Mira A., Collado M.C. (2015). Microbial mucosal colonic shifts associated with the development of colorectal cancer reveal the presence of different bacterial and archaeal biomarkers. J. Gastroenterol..

[35] Thomas A.M., Jesus E.C., Lopes A., Aguiar S., Begnami M.D., Rocha R.M., Carpinetti P.A., Camargo A.A., Hoffmann C., Freitas H.C. (2016). Tissue-Associated Bacterial Alterations in Rectal Carcinoma Patients Revealed by 16S rRNA Community Profiling. Front. Cell Infect. Microbiol..

[36] Zhang H., Chang Y., Zheng Q., Zhang R., Hu C., Jia W. (2019). Altered intestinal microbiota associated with colorectal cancer. Front. Med..

[37] Allali I., Delgado S., Marron P.I., Astudillo A., Yeh J.J., Ghazal H., Amzazi S., Keku T., Azcarate-Peril M.A. (2015). Gut microbiome compositional and functional differences between tumor and non-tumor adjacent tissues from cohorts from the US and Spain. Gut Microbes.

[38] Brim H., Yooseph S., Lee E., Sherif Z.A., Abbas M., Laiyemo A.O., Varma S., Torralba M., Dowd S.E., Nelson K.E. (2017). A Microbiomic Analysis in African Americans with Colonic Lesions Reveals Streptococcus sp.VT162 as a Marker of Neoplastic Transformation. Genes.

[39] Burns M.B., Lynch J., Starr T.K., Knights D., Blekhman R. (2015). Virulence genes are a signature of the microbiome in the colorectal tumor microenvironment. Genome Med..

[40] Chen W., Liu F., Ling Z., Tong X., Xiang C. (2012). Human intestinal lumen and mucosa-associated microbiota in patients with colorectal cancer. PLoS ONE.

[41] Cremonesi E., Governa V., Garzon J.F.G., Mele V., Amicarella F., Muraro M.G., Trella E., Galati-Fournier V., Oertli D., Däster S.R. (2018). Gut microbiota modulate T cell trafficking into human colorectal cancer. Gut.

[42] de Carvalho A.C., de Mattos Pereira L., Datorre J.G., Dos Santos W., Berardinelli G.N., Matsushita M.M., Oliveira M.A., Durães R.O., Guimarães D.P., Reis R.M. (2019). Microbiota Profile and Impact of *Fusobacterium nucleatum* in Colorectal Cancer Patients of Barretos Cancer Hospital. Front. Oncol..

[43] Drewes J.L., White J.R., Dejea C.M., Fathi P., Iyadorai T., Vadivelu J., Roslani A.C., Wick E.C., Mongodin E.F., Loke M.F. (2017). High-resolution bacterial 16S rRNA gene profile meta-analysis and biofilm status reveal common colorectal cancer consortia. NPJ Biofilms Microbiomes.

[44] Gao R., Kong C., Huang L., Li H., Qu X., Liu Z., Lan P., Wang J., Qin H. (2017). Mucosa-associated microbiota signature in colorectal cancer. Eur. J. Clin. Microbiol. Infect. Dis.

[45] Geng J., Fan H., Tang X., Zhai H., Zhang Z. (2013). Diversified pattern of the human colorectal cancer microbiome. Gut Pathog..

[46] Hale V.L., Jeraldo P., Mundy M., Yao J., Keeney G., Scott N., Cheek E.H., Davidson J., Greene M., Martinez C. (2018). Synthesis of multi-omic data and community metabolic models reveals insights into the role of hydrogen sulfide in colon cancer. Methods.

[47] Kinross J., Mirnezami R., Alexander J., Brown R., Scott A., Galea D., Veselkov K., Goldin R., Darzi A., Nicholson J. (2017). A prospective analysis of mucosal microbiome-metabonome interactions in colorectal cancer using a combined MAS 1HNMR and metataxonomic strategy. Sci. Rep..

[48] Leung P.H.M., Subramanya R., Mou Q., Lee K.T., Islam F., Gopalan V., Lu C.T., Lam A.K. (2019). Characterization of Mucosa-Associated Microbiota in Matched Cancer and Non-neoplastic Mucosa From Patients With Colorectal Cancer. Front. Microbiol..

[49] Liu C.J., Zhang Y.L., Shang Y., Wu B., Yang E., Luo Y.Y., Li X.R. (2019). Intestinal bacteria detected in cancer and adjacent tissue from patients with colorectal cancer. Oncol. Lett..

[50] Loke M.F., Chua E.G., Gan H.M., Thulasi K., Wanyiri J.W., Thevambiga I., Goh K.L., Wong W.F., Vadivelu J. (2018). Metabolomics and 16S rRNA sequencing of human colorectal cancers and adjacent mucosa. PLoS ONE.

[51] Pan H.W., Du L.T., Li W., Yang Y.M., Zhang Y., Wang C.X. (2020). Biodiversity and richness shifts of mucosa-associated gut microbiota with progression of colorectal cancer. Res. Microbiol..

[52] Saffarian A., Mulet C., Regnault B., Amiot A., Tran-Van-Nhieu J., Ravel J., Sobhani I., Sansonetti P.J., Pédron T. (2019). Crypt- and Mucosa-Associated Core Microbiotas in Humans and Their Alteration in Colon Cancer Patients. mBio.

[53] Sheng Q.S., He K.X., Li J.J., Zhong Z.F., Wang F.X., Pan L.L., Lin J.J. (2020). Comparison of Gut Microbiome in Human Colorectal Cancer in Paired Tumor and Adjacent Normal Tissues. Onco Targets Ther..

[54] Wang Q., Ye J., Fang D., Lv L., Wu W., Shi D., Li Y., Yang L., Bian X., Wu J. (2020). Multi-omic profiling reveals associations between the gut mucosal microbiome, the metabolome, and host DNA methylation associated gene expression in patients with colorectal cancer. BMC Microbiol..

[55] Wirth U., Garzetti D., Jochum L.M., Spriewald S., Kühn F., Ilmer M., Lee S.M.L., Niess H., Bazhin A.V., Andrassy J. (2020). Microbiome Analysis from Paired Mucosal and Fecal Samples of a Colorectal Cancer Biobank. Cancers.

[56] Zeller G., Tap J., Voigt A.Y., Sunagawa S., Kultima J.R., Costea P.I., Amiot A., Böhm J., Brunetti F., Habermann N. (2014). Potential of fecal microbiota for early-stage detection of colorectal cancer. Mol. Syst. Biol..

[57] Flemer B., Lynch D.B., Brown J.M., Jeffery I.B., Ryan F.J., Claesson M.J., O’Riordain M., Shanahan F., O’Toole P.W. (2017). Tumour-associated and non-tumour-associated microbiota in colorectal cancer. Gut.

[58] Gao Z., Guo B., Gao R., Zhu Q., Qin H. (2015). Microbiota disbiosis is associated with colorectal cancer. Front. Microbiol..

[59] Nakatsu G., Li X., Zhou H., Sheng J., Wong S.H., Wu W.K., Ng S.C., Tsoi H., Dong Y., Zhang N. (2015). Gut mucosal microbiome across stages of colorectal carcinogenesis. Nat. Commun..

[60] Richard M.L., Liguori G., Lamas B., Brandi G., da Costa G., Hoffmann T.W., Pierluigi Di Simone M., Calabrese C., Poggioli G., Langella P. (2018). Mucosa-associated microbiota dysbiosis in colitis associated cancer. Gut Microbes.

[61] Wang Y., Zhang C., Hou S., Wu X., Liu J., Wan X. (2020). Analyses of Potential Driver and Passenger Bacteria in Human Colorectal Cancer. Cancer Manag. Res..

[62] Choi S., Chung J., Cho M.L., Park D., Choi S.S. (2021). Analysis of changes in microbiome compositions related to the prognosis of colorectal cancer patients based on tissue-derived 16S rRNA sequences. J. Transl. Med..

[63] Liu W., Zhang X., Xu H., Li S., Lau H.C., Chen Q., Zhang B., Zhao L., Chen H., Sung J.J. (2021). Microbial Community Heterogeneity Within Colorectal Neoplasia and its Correlation With Colorectal Carcinogenesis. Gastroenterology.

[64] Malik S.A., Zhu C., Li J., LaComb J.F., Denoya P.I., Kravets I., Miller J.D., Yang J., Kramer M., McCombie W.R. (2021). Impact of preoperative antibiotics and other variables on integrated microbiome-host transcriptomic data generated from colorectal cancer resections. World J. Gastroenterol..

[65] Nardelli C., Granata I., Nunziato M., Setaro M., Carbone F., Zulli C., Pilone V., Capoluongo E.D., De Palma G.D., Corcione F. (2021). 16S rRNA of Mucosal Colon Microbiome and CCL2 Circulating Levels Are Potential Biomarkers in Colorectal Cancer. Int. J. Mol. Sci..

[66] Niccolai E., Russo E., Baldi S., Ricci F., Nannini G., Pedone M., Stingo F.C., Taddei A., Ringressi M.N., Bechi P. (2020). Significant and Conflicting Correlation of IL-9 With *Prevotella* and *Bacteroides* in Human Colorectal Cancer. Front. Immunol..

[67] Okuda S., Shimada Y., Tajima Y., Yuza K., Hirose Y., Ichikawa H., Nagahashi M., Sakata J., Ling Y., Miura N. (2021). Profiling of host genetic alterations and intra-tumor microbiomes in colorectal cancer. Comput. Struct. Biotechnol. J..

[68] Osman M.A., Neoh H.M., Ab Mutalib N.S., Chin S.F., Mazlan L., Raja Ali R.A., Zakaria A.D., Ngiu C.S., Ang M.Y., Jamal R. (2021). *Parvimonas micra*, *Peptostreptococcus stomatis*, *Fusobacterium nucleatum* and *Akkermansia muciniphila* as a four-bacteria biomarker panel of colorectal cancer. Sci. Rep..

[69] Wang Y., Zhang Y., Qian Y., Xie Y.H., Jiang S.S., Kang Z.R., Chen Y.X., Chen Z.F., Fang J.Y. (2021). Alterations in the oral and gut microbiome of colorectal cancer patients and association with host clinical factors. Int. J. Cancer.

[70] Zhang J., Tao J., Gao R.N., Wei Z.Y., He Y.S., Ren C.Y., Li Q.C., Liu Y.S., Wang K.W., Yang G. (2021). Cytotoxic T-Cell Trafficking Chemokine Profiles Correlate With Defined Mucosal Microbial Communities in Colorectal Cancer. Front. Immunol..

[71] Tilg H., Kaser A. (2011). Gut microbiome, obesity, and metabolic dysfunction. J. Clin. Investig..

[72] Ahn J., Sinha R., Pei Z., Dominianni C., Wu J., Shi J., Goedert J.J., Hayes R.B., Yang L. (2013). Human gut microbiome and risk for colorectal cancer. J. Natl. Cancer Inst..

[73] Zhang B., Xu S., Xu W., Chen Q., Chen Z., Yan C., Fan Y., Zhang H., Liu Q., Yang J. (2019). Leveraging fecal bacterial survey data to predict colorectal tumors. Front. Genet..

[74] Yang Y., Misra B.B., Liang L., Bi D., Weng W., Wu W., Cai S., Qin H., Goel A., Li X. (2019). Integrated microbiome and metabolome analysis reveals a novel interplay between commensal bacteria and metabolites in colorectal cancer. Theranostics.

[75] Flanagan L., Schmid J., Ebert M., Soucek P., Kunicka T., Liska V., Bruha J., Neary P., Dezeeuw N., Tommasino M. (2014). *Fusobacterium nucleatum* associates with stages of colorectal neoplasia development, colorectal cancer and disease outcome. Eur. J. Clin. Microbiol. Infect. Dis..

[76] Mima K., Nishihara R., Qian Z.R., Cao Y., Sukawa Y., Nowak J.A., Yang J., Dou R., Masugi Y., Song M. (2016). *Fusobacterium nucleatum* in colorectal carcinoma tissue and patient prognosis. Gut.

[77] Yu T., Guo F., Yu Y., Sun T., Ma D., Han J., Qian Y., Kryczek I., Sun D., Nagarsheth N. (2017). *Fusobacterium nucleatum* promotes chemoresistance to colorectal cancer by modulating autophagy. Cell.

[78] Rubinstein M.R., Wang X., Liu W., Hao Y., Cai G., Han Y.W. (2013). *Fusobacterium nucleatum* promotes colorectal carcinogenesis by modulating E-cadherin/beta-catenin signaling via its FadA adhesin. Cell Host Microbe.

[79] He Z., Gharaibeh R.Z., Newsome R.C., Pope J.L., Dougherty M.W., Tomkovich S., Pons B., Mirey G., Vignard J., Hendrixson D.R. (2019). Campylobacter jejuni promotes colorectal tumorigenesis through the action of cytolethal distending toxin. Gut.

[80] Abdulamir A.S., Hafidh R.R., Bakar F.A. (2011). The association of Streptococcus bovis/gallolyticus with colorectal tumors: The nature and the underlying mechanisms of its etiological role. J. Exp. Clin. Cancer Res..

[81] Abdulamir A.S., Hafidh R.R., Bakar F.A. (2010). Molecular detection, quantification, and isolation of Streptococcus gallolyticus bacteria colonizing colorectal tumors: Inflammation-driven potential of carcinogenesis via IL-1, COX-2, and IL-8. Mol. Cancer.

[82] Edwards A.M., Grossman T.J., Rudney J.D. (2006). *Fusobacterium nucleatum* transports noninvasive *Streptococcus cristatus* into human epithelial cells. Infect. Immun..

[83] Warren R.L., Freeman D.J., Pleasance S., Watson P., Moore R.A., Cochrane K., Allen-Vercoe E., Holt R.A. (2013). Co-occurrence of anaerobic bacteria in colorectal carcinomas. Microbiome.

[84] Yu J., Zhao L., Zhao R., Long X., Coker O.O., Sung J.J. (2019). The role of *Parvimonas micra* in intestinal tumorigenesis in germ-free and conventional APCmin/+ mice. J. Clin. Oncol..

[85] Long X., Wong C.C., Tong L., Chu E.S., Ho Szeto C., Go M.Y., Coker O.O., Chan A.W., Chan F.K., Sung J.J. (2019). *Peptostreptococcus anaerobius* promotes colorectal carcinogenesis and modulates tumour immunity. Nat. Microbiol..

[86] Tsoi H., Chu E.S., Zhang X., Sheng J., Nakatsu G., Ng S.C., Chan A.W., Chan F.K., Sung J.J., Yu J. (2017). *Peptostreptococcus anaerobius* induces intracellular cholesterol biosynthesis in colon cells to induce proliferation and causes dysplasia in mice. Gastroenterology.

[87] Boleij A., Hechenbleikner E.M., Goodwin A.C., Badani R., Stein E.M., Lazarev M.G., Ellis B., Carroll K.C., Albesiano E., Wick E.C. (2014). The *Bacteroides fragilis* Toxin Gene Is Prevalent in the Colon Mucosa of Colorectal Cancer Patients. Clin. Infect. Dis..

[88] Sanfilippo L., Li C., Seth R., Balwin T., Menozzi M., Mahida Y. (2000). Bacteroides fragilis enterotoxin induces the expression of IL-8 and transforming growth factor-beta (TGF-β) by human colonic epithelial cells. Clin. Exp. Immunol..

[89] Ferreira-Halder C.V., de Sousa Faria A.V., Andrade S.S. (2017). Action and function of *Faecalibacterium prausnitzii* in health and disease. Best Pract. Res. Clin. Gastroenterol..

[90] Zhang M., Qiu X., Zhang H., Yang X., Hong N., Yang Y., Chen H., Yu C. (2014). *Faecalibacterium prausnitzii* inhibits interleukin-17 to ameliorate colorectal colitis in rats. PLoS ONE.

[91] Koh G.Y., Kane A.V., Wu X., Crott J.W. (2020). Parabacteroides distasonis attenuates tumorigenesis, modulates inflammatory markers and promotes intestinal barrier integrity in azoxymethane-treated A/J mice. Carcinogenesis.

[92] Liu X., Mao B., Gu J., Wu J., Cui S., Wang G., Zhao J., Zhang H., Chen W. (2021). Blautia—A new functional genus with potential probiotic properties?. Gut Microbes.

[93] Jones S., Chen W.D., Parmigiani G., Diehl F., Beerenwinkel N., Antal T., Traulsen A., Nowak M.A., Siegel C., Velculescu V.E. (2008). Comparative lesion sequencing provides insights into tumor evolution. Proc. Natl. Acad. Sci. USA.

[94] Zackular J.P., Baxter N.T., Iverson K.D., Sadler W.D., Petrosino J.F., Chen G.Y., Schloss P.D. (2013). The gut microbiome modulates colon tumorigenesis. mBio.

[95] Tjalsma H., Boleij A., Marchesi J.R., Dutilh B.E. (2012). A bacterial driver–passenger model for colorectal cancer: Beyond the usual suspects. Nat. Rev. Microbiol..

[96] Suzuki T.A., Ley R.E. (2020). The role of the microbiota in human genetic adaptation. Science.

[97] Knight R., Vrbanac A., Taylor B.C., Aksenov A., Callewaert C., Debelius J., Gonzalez A., Kosciolek T., McCall L.I., McDonald D. (2018). Best practices for analysing microbiomes. Nat. Rev. Microbiol..

[98] Bukin Y.S., Galachyants Y.P., Morozov I.V., Bukin S.V., Zakharenko A.S., Zemskaya T.I. (2019). The effect of 16S rRNA region choice on bacterial community metabarcoding results. Sci. Data.

[99] Graf D., Di Cagno R., Fåk F., Flint H.J., Nyman M., Saarela M., Watzl B. (2015). Contribution of diet to the composition of the human gut microbiota. Microb. Ecol. Health Dis..

