# Supplementary material for: The Tissue-Associated Microbiota in Colorectal Cancer: A Systematic Review
Source: Cancers (Basel). 2022 Jul 12;14(14):3385. doi: 10.3390/cancers14143385 (PMC9317273; doi:10.3390/cancers14143385)
Supplement: Supplementary file 1 [file cancers-14-03385-s001.zip › cancers-1719014-supplementary.pdf]

SUPPLEMENTARY TABLES

Systematic Review

The tissue-associated microbiota in colorectal cancer: a systematic review

Carolina P. Costa 1,2,3,†, Patrícia Vieira 1,2,3,†, Melissa Mendes-Rocha 1,2,3, Joana Pereira-Marques 1,2, Rui M. Ferreira 1,2 and Ceu Figueiredo 1,2,3,*

1 Instituto de Investigação e Inovação em Saúde, Universidade do Porto (i3S), Porto, Portugal;
2 Institute of Molecular Pathology and Immunology of the University of Porto (IPATIMUP), Porto, Portugal;
3 Department of Pathology, Faculty of Medicine of the University of Porto, Porto, Portugal.
† These authors contributed equally to this work.
* Correspondence: cfigueiredo@ipatimup.pt

Supplementary Table S1. Microbiome methodological analysis of included studies of the mucosal microbiota of CRC patients and healthy controls.
Supplementary Table S2. Microbiome methodological analysis of included studies of CRC and non-cancerous mucosal tissues.
Supplementary Table S3. Quality of included CRC vs HC studies based on the Newcastle-Ottawa quality assessment scale.
Supplementary Table S4. Quality of included CRC vs NCT studies based on the Newcastle-Ottawa quality assessment scale.
Supplementary Table S5. Summary of the statistically significant microbial taxa identified in studies comparing CRC vs HC and CRC vs NCT.
Supplementary Table S6. Qualitative synthesis showing the strong and the suggestive microbial taxonomic associations with CRC.

Note: Reference numbers can be found in the reference list of the main manuscript.

Supplementary Table S1. Microbiome methodological analysis of included studies of the mucosal microbiota of CRC patients and healthy controls.

First author, year	16S rRNA gene target region	Sequencing platform	Database for taxonomy assignment (version)	Statistical analysis for taxonomic differences
**Geng, 2014 [33]**	V1-V2	454 GS FLX (Roche)	NR	Univariate analysis: ANOVA and Kruskal-Wallis
**Gao, 2015 [58]**	V3	454 GS FLX (Roche)	RDP (11.1), SILVA (115), Greengenes (13.5)	LEfSe analysis
**Mira-Pascual, 2015 [34]**	V1-V3	454 GS FLX (Roche)	RDP (NR)	NR
**Nakatsu, 2015 [59]**	V1-V4	454 GS FLX (Roche)	Greengenes (13.8)	LEfSe analysis and Mann-Whitney U-test
**Thomas, 2016 [35]**	V4-V5	Ion Torrent PGM	NR	Wilcoxon rank sum test
**Flemer, 2017 [57]**	V3-V4	Illumina MiSeq	RDP (14)	Student t-test and Wilcoxon rank sum test
**Richard, 2018 [60]**	V3-V4	Illumina MiSeq	Greengenes (NR)	LEfSe analysis and One-way ANOVA
**Zhang, 2019 [36]**	V6	Illumina MiSeq	Greengenes (13.5)	LEfSe analysis and Mann-Whitney U-test
**Wang Y, 2020 [61]**	V3-V4	Illumina MiSeq	NR	Kruskal–Wallis test
**Nardelli, 2021 [65]**	V3-V6	Illumina MiSeq	16S-UDb (NR)	DESeq2
**Osman, 2021 [68]**	V3-V4	Illumina MiSeq	Greengenes (13.5) and One Codex Targeted Loci database (NR)	LEfSe analysis and Mann-Whitney U-test
**Wang, 2021 [69]**	NR	Illumina MiSeq	SILVA (132)	Benjamini-Hochberg false discovery rate

Abbreviations: CRC: colorectal cancer; LEfSe: Linear discriminant analysis Effect Size; NR: not reported; RDP – Ribosomal Database Project; 16S-UDb – unified 16S rRNA database.

Supplementary Table S2. Microbiome methodological analysis of included studies of CRC and non-cancerous mucosal tissues.

First author, year	16S rRNA gene target region	Sequencing platform	Database for taxonomy assignment (version)	Statistical analysis for taxonomic differences
**Marchesi, 2011 [19]**	V1-V3	454 GS FLX (Roche)	RDP (NR)	NR
**Chen, 2012 [40]**	V1-V3	454 GS FLX (Roche)	SILVA (106)	LEfSe analysis
**Geng, 2013 [45]**	V1-V2	454 GS FLX (Roche)	BLAST (NR)	Mann-Whitney U-test and Student t-test
**Zeller, 2014 [56]**	V4	Illumina MiSeq	SILVA (115)	Wilcoxon signed rank test
**Allali, 2015 [37]**	V1-V2	454 GS FLX (Roche)	Greengenes (NR)	Wilcoxon signed rank test
**Burns, 2015 [39]**	V5-V6	Illumina MiSeq	Greengenes (NR)	Wilcoxon signed rank test
**Gao, 2015 [58]**	V3	454 GS FLX (Roche)	RDP (11.1), SILVA (115), Greengenes (13.5)	NR
**Nakatsu, 2015 [59]**	V1-V4	454 GS FLX (Roche)	Greengenes (13.8)	LEfSe analysis and Mann-Whitney U-test
**Brim, 2017 [38]**	V1-V3	454 GS FLX (Roche)	NR	NR
**Drewes, 2017 [43]**	V3-V4	Illumina MiSeq	Greengenes (13.05)	Hedge’s g difference statistic
**Flemer, 2017 [57]**	V3-V4	Illumina MiSeq	RDP (14)	Student t-test and Wilcoxon signed rank test
**Gao, 2017 [44]**	V4	Illumina MiSeq	NR	LEfSe analysis
**Kinross, 2017 [47]**	V1-V3	454 GS FLX (Roche)	NR	NR
**Cremonesi, 2018 [41]**	V4	Illumina MiSeq	SILVA (128)	Wilcoxon signed rank test
**Hale, 2018 [46]**	V3-V5	Illumina MiSeq	NR	Kruskal-Wallis test
**Loke, 2018 [50]**	V3-V4	Illumina MiSeq	NCBI 16S microbial database (NR)	Wilcoxon signed rank test
**Richard, 2018 [60]**	V3-V4	Illumina MiSeq	Greengenes (NR)	LEfSe analysis and One-way ANOVA
**de Carvalho, 2019 [42]**	V4	Ion Torrent PGM	Greengenes (13.8)	DESeq2

**Leung, 2019 [48]**	V3-V4	Illumina HiSeq	NR	Wilcoxon signed rank test, Mann-Whitney U-test, Kruskal-Wallis test and LEfSe analysis
**Liu, 2019 [49]**	V3-V4	Illumina MiSeq	SILVA (123)	NR
**Saffarian, 2019 [52]**	V3-V4	Illumina MiSeq	Greengenes (13.8) and HITdb (NR)	Kruskal-Wallis test and Zero-inflated Gaussian mixture model
**Pan, 2020 [51]**	V4	Illumina MiSeq	SILVA (NR)	LEfSe analysis
**Sheng, 2020 [53]**	V3-V4	Illumina MiSeq	NR	Mann-Whitney U-test
**Wang Q, 2020 [54]**	V3-V4	Ion Torrent S5TM XL	SILVA (123)	Paired zero-inflated Gaussian mixture model
**Wang Y, 2020 [61]**	V3-V4	Illumina MiSeq	NR	Kruskal–Wallis test
**Wirth, 2020 [55]**	V3-V4	Illumina MiSeq	SILVA (132)	Wilcoxon rank sum test and Friedman two-way analysis of variance
**Choi, 2021 [62]**	V3-V4	Illumina MiSeq	Greengenes (13.8)	LEfSe analysis and Student t-test
**Liu, 2021 [63]**	V4	NR	Greengenes (NR)	MaAsLin analysis
**Malik, 2021 [64]**	V3-V4	Illumina MiSeq	SILVA (115)	Linear mixed models and Student t-test
**Nardelli, 2021 [65]**	V3-V6	Illumina MiSeq	16S-UDb (NR)	DESeq2
**Niccolai, 2021 [66]**	V3-V4	Illumina MiSeq	NR	DESeq2
**Okuda, 2021 [67]**	NR	Illumina MiSeq	NR	Wilcoxon rank sum test
**Zhang, 2021 [70]**	V4	NR	Greengenes (NR)	Wilcoxon signed rank test with false discovery rate

Abbreviations: HITdb: human intestinal 16S rRNA gene taxonomic database; LEfSe: Linear discriminant analysis effect size; MaAsLin: Microbiome multivariable association with linear models; NR: not reported; RDP: Ribosomal Database Project; 16S-UDb – unified 16S rRNA database.

Supplementary Table S3. Quality of included CRC vs HC studies based on the Newcastle-Ottawa quality assessment scale.

First author, year	Selection	Comparability	Outcome	Total (out of 9)
**Geng, 2014 [33]**	★☆★★	☆☆	☆★★	5
**Gao, 2015 [58]**	★☆★★	★★	★★★	8
**Mira-Pascual, 2015 [34]**	★★★★	★★	☆★☆	7
**Nakatsu, 2015 [59]**	★★★★	★★	☆★★	8
**Thomas, 2016 [35]**	★☆★★	★★	★★★	8
**Flemer, 2017 [57]**	★☆☆★	★★	★★★	7
**Richard, 2018 [60]**	★☆★★	★☆	★★★	7
**Zhang, 2019 [36]**	★☆☆☆	☆★	★★☆	4
**Wang Y, 2020 [61]**	★☆☆☆	☆☆	☆★★	3
**Nardelli, 2021 [65]**	★☆☆★	★☆	★★★	6
**Osman, 2021 [68]**	★★★★	★★	★★☆	8
**Wang, 2021 [69]**	★☆☆★	☆☆	☆★☆	3

Abbreviations: CRC: colorectal cancer; HC: healthy controls.

Supplementary Table S4. Quality of included CRC vs NCT studies based on the Newcastle-Ottawa quality assessment scale.

First author, year	Selection	Comparability	Outcome	Total (out of 9)
**Marchesi, 2011 [19]**	★☆★★	★★	☆★☆	6
**Chen, 2012 [40]**	★☆★★	★★	★★★	8
**Geng, 2013 [45]**	★☆★★	★★	☆★☆	6
**Zeller, 2014 [56]**	★☆★★	★★	☆★★	7
**Allali, 2015 [37]**	★☆★★	★★	☆★★	7
**Burns, 2015 [39]**	★☆★★	★★	☆★★	7
**Gao, 2015 [58]**	★☆★★	★★	★★☆	7
**Nakatsu, 2015 [59]**	★★★★	★★	☆★★	8
**Brim, 2017 [38]**	★☆★★	★★	☆★☆	6
**Drewes, 2017 [43]**	★☆★★	★★	☆★★	7
**Flemer, 2017 [57]**	★☆★★	★★	★★★	8
**Gao, 2017 [44]**	★☆★★	★★	★★☆	7
**Kinross, 2017 [47]**	★☆★★	★★	★★☆	7
**Cremonesi, 2018 [41]**	★☆★★	★★	☆★☆	6
**Hale, 2018 [46]**	★★★★	★★	☆★☆	7
**Loke, 2018 [50]**	★★★★	★★	☆★★	8
**Richard, 2018 [60]**	★☆★★	★★	★★★	8
**de Carvalho, 2019 [42]**	★★★★	★★	☆★☆	7
**Leung, 2019 [48]**	★★★★	★★	☆★★	8
**Liu, 2019 [49]**	★☆★★	★★	☆★☆	6
**Saffarian, 2019 [52]**	★☆★★	★★	★★☆	7
**Pan, 2020 [51]**	★☆★★	★★	★★★	8
**Sheng, 2020 [53]**	★★★★	★★	★★☆	8
**Wang Q, 2020 [54]**	★☆★★	★★	★★★	8
**Wang Y, 2020 [61]**	★☆★★	★★	☆★★	7
**Wirth, 2020 [55]**	★☆★★	★★	☆★★	7
**Choi, 2021 [62]**	★☆★★	★★	☆★★	7
**Liu, 2021 [63]**	★☆★★	★★	☆★★	7
**Malik, 2021 [64]**	★★★★	★★	★★★	9
**Nardelli, 2021 [65]**	★☆★★	★★	★★★	8
**Niccolai, 2021 [66]**	★☆★★	★★	★★★	8
**Okuda, 2021 [67]**	★☆★★	★★	☆★★	7
**Zhang, 2021 [70]**	★★★★	★★	★★☆	8

Abbreviations: CRC: colorectal cancer; NCT: non-cancerous tissue.

Supplementary Table S5. Summary of the statistically significant microbial taxa identified in studies comparing CRC vs HC and CRC vs NCT.

		CRC vs HC		CRC vs NCT	
	**Microbial taxa**	**Increased in HC**	**Increased in CRC**	**Increased in NCT**	**Increased in CRC**
**Phylum**	Acidobacteria	-	-	1 [70]	0
	AC1	-	-	1 [70]	0
	Actinobacteria	2 [35,65]	0	4 [44,55,62,70]	0
	Armatimonadetes	-	-	1 [70]	0
	Bacteroidetes	2 [59,60]	2 [35,57]	5 [39,41,60,62,64]	0
	Chlorobi	-	-	1 [70]	0
	Chloroflexi	-	-	1 [70]	0
	Cyanobacteria	1 [35]	0	2 [64,70]	0
	Deferribacteres	-	-	1 [70]	0
	Elusimicrobia	-	-	1 [70]	0
	WPS-2 (Eremiobacterota)	-	-	1 [70]	0
	Fibrobacteres	-	-	1 [70]	0
	Firmicutes	0	1 [58]	5 [39,41,44,62]	1 [58]
	Fusobacteria	0	4 [58,60,65,68]	0	8 [37,39,41,44,48,62,66,70]
	Gemmatimonadetes	-	-	1 [70]	0
	GN02 (Gracilibacteria)	-	-	1 [70]	0
	GOUTA4	-	-	1 [70]	0
	Nitrospirae	-	-	1 [70]	0
	OD1 (Parcubacteria)	0	1 [35]	1 [70]	0
	OP3 (Omnitrophica)	-	-	1 [70]	0
	Planctomycetes	1 [35]	0	1 [70]	0
	Proteobacteria	2 [35,58]	1 [60]	1 [58]	3 [39,54,66]
	TM7 (Saccharibacteria)	-	-	1 [70]	0
	SR1	-	-	1 [70]	0
	BRC1 (Sumerlaeota)	-	-	1 [70]	0
	Synergitetes	0	1 [68]	0	1 [70]
	Verrucomicrobia	0	1 [68]	1 [70]	0
	WS4	-	-	1 [70]	0
**Class**	*Alphaproteobacteria*	0	2 [36,68]	0	1 [40]
	Bacilli	-	-	0	2 [40,60]
	Bacteroidia	-	-	2 [60,62]	0
	Betaproteobacteria	1 [68]	0	1 [62]	0
	Deltaproteobacteria	1 [36]	0	-	-
	Epsilonproteobacteria	0	1 [68]	0	1 [62]
	Erysipelotrichi	-	-	0	1 [60]
	Fusobacteriia	-	-	0	1 [62]
	Gammaproteobacteria	1 [35]	0	-	-
	Spirochaetes	-	-	0	1 [62]
	Synergistia	0	1 [68]	-	-
	ZB2	0	1 [35]	-	-
**Order**	*Acidobacteriales*	-	-	1 [70]	0
	Actinomycetales	-	-	1 [70]	0
	Bacillales	1 [59]	0	0	1 [70]
	Bacteroidales	-	-	2 [60,62]	0
	Bifidobacteriales	-	-	2 [62,70]	0
	Burkholderiales	1 [68]	0	2 [62,70]	0

	Campylobacteriales	-	-	0	2 [62,70]
	Clostridiales	0	2 [35,57]	2 [62,64]	-
	Coriobacteriia	-	-	1 [62]	0
	Desulfovibrionales	1 [36]	0	-	-
	Erysipelotrichales	-	-	1 [70]	1 [60]
	Flavobacteriales	-	-	1 [70]	0
	Fusobacteriales	0	1 [68]	0	2 [62,70]
	GCA004	-	-	0	1 [70]
	Gemellales	-	-	0	1 [70]
	Gemmatimonadales	-	-	1 [70]	0
	Lactobacillales	-	-	0	1 [60]
	Mycoplasmatales	-	-	0	1 [70]
	Myxococcales	-	-	1 [70]	0
	Phycisphaerales	1 [35]	0	-	-
	Pseudomonadales	-	-	1 [70]	0
	Rhizobiales	0	1 [36]	1 [70]	0
	Rickettsiales	-	-	1 [64]	0
	Sphingobacteriales	1 [35]	0	1 [70]	0
	Spirochaetales	-	-	0	1 [62]
	Streptophyta	-	-	1 [70]	0
	Verrucomicrobiales	0	1 [68]	-	-
	Xanthomonadales	-	-	0	1 [70]
**Family**	*0319-6A21*	-	-	0	1 [63]
	Alcaligenaceae	1 [68]	0	1 [62]	0
	Bifidobacteriaceae	-	-	2 [62,70]	0
	Bradyrhizobiaceae	1 [35]	0	1 [70]	0
	Campylobacteriaceae	0	1 [68]	0	2 [62,70]
	Carnobacteriaceae	-	-	0	1 [64]
	Caulobacteraceae	1 [35]	0	-	-
	Chitinophagaceae	1 [35]	0	1 [70]	0
	Christensenellaceae	0	1 [68]	-	-
	Clostridiaceae	1 [36]	0	-	-
	Comamonadaceae	-	-	1 [70]	0
	Coriobacteriaceae	-	-	2 [62,64]	1 [37]
	Desulfovibrionaceae	1 [36]	0	-	-
	Enterobacteriaceae	-	-	0	1 [42]
	Erysipelotrichaceae	-	-	2 [62,70]	1 [60]
	Flavobacteriaceae	1 [35]	0	1 [70]	0
	Fusobacteriaceae	0	1 [68]	0	3 [42,62,70]
	Gemelllaceae	-	-	0	2 [67,70]
	Halomonadaceae	-	-	0	1 [54]
	Hyphomicrobiaceae	0	1 [36]	-	-
	Intrasporangiaceae	1 [61]	0	1 [61]	1 [70]
	Koribacteraceae	-	-	1 [70]	0
	Lachnospiraceae	0	1 [35]	3 [39,62,64]	1 [37]
	Listeriaceae	-	-	1 [70]	0
	Micrococcaceae	-	-	0	1 [70]
	Mogibacteriaceae	0	1 [35]	-	-
	Moraxellaceae	-	-	1 [70]	0
	Mycoplasmataceae	-	-	0	1 [70]
	Muribaculaceae	-	-	2 [64,70]	0

	Neisseriaceae	-	-	0	1 [37]
	Nocardioidaceae	-	-	0	1 [70]
	Odoribacteraceae	0	1 [35]	1 [62]	0
	Oxalobacteraceae	-	-	1 [62]	0
	Paenibacillaceae	0	1 [36]	-	-
	Peptostreptococcaceae	-	-	0	1 [63]
	Phyllobacteriaceae	-	-	1 [70]	0
	Porphyromonadaceae	0	1 [57]	4 [37,60,62,70]	0
	Prevotellaceae	1 [36]	0	-	-
	Pseudomonadaceae	-	-	1 [70]	0
	Rikenellaceae	0	1 [35]	4 [39,46,67,70]	0
	Ruminococcaceae	0	1 [35]	7 [39,40,53,60,62,67,70]	0
	Shewanellaceae	-	-	0	1 [70]
	Sinobacteraceae	-	-	1 [61]	0
	Sphingomonadaceae	-	-	1 [70]	0
	Spirochaetaceae	-	-	0	1 [62]
	Streptococcaceae	0	1 [33]	0	2 [42,60]
	Streotomycetaceae	-	-	0	1 [70]
	Syntrophobacteraceae	-	-	0	1 [63]
	Tissierellaceae	0	1 [68]	-	-
	Verrucomicrobiaceae	0	1 [68]	-	-
	Xanthomonadaceae	1 [35]	0	0	1 [70]
**Genus**	*Acetanaerobacterium*	0	1 [57]	-	-
	Achromobacter	1 [35]	0	-	-
	Acidocella	-	-	1 [40]	0
	Acidovorax	1 [58]	0	-	-
	Acinetobacter	2 [35,58]	0	3 [44,52,70]	0
	Actinomyces	0	1 [57]	1 [50]	0
	Aeromonas	-	-	0	1 [42]
	Aggregatibacter	0	1 [59]	-	-
	Agrobacterium	1 [35]	0	-	-
	Akkermansia	0	1 [68]	3 [53,64,67]	0
	Alistipes	0	1 [57]	1 [50]	0
	Allisonella	0	1 [57]	-	-
	Allobaculum	-	-	1 [64]	0
	Alloprevotella	-	-	0	1 [44]
	Anaerococcus	1 [35]	0	0	1 [61]
	Anaerofustis	1 [68]	0	-	-
	Anaeroplasma	-	-	1 [64]	0
	Anaerorhabdus	1 [57]	0	-	-
	Anaerostipes	1 [57]	1 [35]	-	-
	Anaerotruncus	0	1 [35]	1 [50]	0
	Anoxybacillus	-	-	1 [45]	0
	Arthrobacter	-	-	0	1 [70]
	Bacillus	1 [35]	0	3 [40,44,61]	0
	Bacteroides	1 [59]	2 [35,57]	5 [39,42,46,50,67]	1 [58]
	Bacteroidetes_VC2_I_Bac22	1 [35]	0	-	-
	Bifidobacterium	0	1 [57]	3 [50,62,70]	0
	Bilophila	0	1 [35]	-	-
	Blautia	3 [57,59,68]	0	4 [37,46,59,60]	0
	Bosea	1 [35]	0	-	-

Bradyrhizobium	-	-	2 [61,70]	0
Brevibacterium	1 [35]	0	-	-
Brevundimonas	2 [35,58]	0	1 [48]	0
Brochothrix	-	-	1 [70]	0
Bulleidia	0	1 [68]	0	1 [37]
Burkholderia	-	-	0	1 [63]
Butyricimonas	0	1 [35]	1 [67]	1 [37]
Butyricoccus	0	1 [35]	-	-
Buttiauxella	1 [58]	0	-	-
Campylobacter	0	4 [57,59,61,68]	0	9 [37,42,44,48,61,62,64,67,70]
Candidatus	-	-	0	1 [39]
Catenibacterium	-	-	0	1 [64]
Catonella	-	-	0	1 [70]
Caulobacter	1 [58]	0	-	-
Cetobacterium	-	-	0	1 [42]
Cheryseobacterium	0	1 [36]	-	-
Chitinophaga	1 [35]	0	-	-
Citrobacter	-	-	0	1 [64]
02d06	1 [36]	0	-	-
Clostridium	0	1 [35]	2 [67,70]	1 [42]
Clostridium cluster IV	0	1 [57]	-	-
Clostridum cluster XIVa	1 [57]	0	0	0
Clostridium cluster XVIII	1 [57]	0	-	-
Christensenella	-	-	1 [50]	0
Collinsella	-	-	3 [50,62,67]	0
Coprococcus	1 [57]	0	-	-
Corynebaterium	1 [35]	0	-	-
Cryocola	1 [35]	0	-	-
Cupriavidus	1 [35]	0	-	-
Delftia	1 [35]	0	-	-
Dehalobacterium	0	2 [35,68]	-	-
Desulfovibrio	0	1 [35]	1 [50]	0
Dethiosulfatibacter	0	1 [61]	-	-
Devosia	1 [35]	1 [36]	-	-
Dialister	0	1 [68]	1 [50]	2 [44,64]
Dorea	2 [57,68]	1 [35]	1 [62]	0
Eggerthella	0	1 [35]	-	-
Eikenella	0	1 [61]	0	2 [37,67]
Enterococcus	0	1 [57]	1 [54]	2 [63,67]
Epilithonimonas	1 [58]	0	-	-
Escherichia	1 [35]	0	-	-
Escherichia-Shigella	0	2 [57,58]	-	-
Eubacterium	2 [36,68]	1 [57]	1 [62]	1 [52]
Faecalibacterium	2 [57,68]	0	5 [37,40,53,60,62]	0
Filifactor	0	1 [68]	-	-
Flavobacterium	1 [58]	0	-	-
Fretibacterium	-	-	0	1 [54]
Fusobacterium	0	6 [35,57-59,61,68]	0	19 [37,39,42,44,46-48,52-54,58,59,61-64,66,67,70]
Gemella	0	1 [59]	0	3 [37,44,59]
Gemmiger	1 [57]	0	-	-

Geobacillus	-	-	1 [44]	0
Gordonia	1 [35]	0	-	-
Granulicatella	0	3 [57,59,61]	0	3 [37,44,59]
Halomonas	-	-	0	1 [54]
Holdemania	1 [57]	1 [35]	-	-
Howardella	0	1 [57]	-	-
Hungatella	-	-	0	1 [44]
Intestinimonas	-	-	1 [50]	0
Janthinobacterium	1 [58]	0	-	-
Kaistobacter	-	-	1 [70]	0
Klebsiella	3 [35,36,57]	0	0	1 [54]
Lachnoanaerobaculum	-	-	0	2 [44,70]
Lachnobacterium	0	1 [68]	-	-
Lachnospira	0	1 [35]	1 [37]	0
Lactobacillus	1 [35]	0	1 [54]	0
Lactococcus	0	1 [58]	1 [44]	1 [58]
Leptothrix	1 [35]	0	0	1 [44]
Leptotrichia	1 [36]	2 [59,61]	1 [42]	2 [64,67]
Leuconostoc	-	-	1 [44]	0
Marvinbryantia	-	-	1 [64]	0
Megasphaera	-	-	2 [50,63]	0
Massilia	-	-	0	1 [52]
Methylobacterium	1 [35]	0	2 [40,61]	0
Mesorhizobium	1 [35]	0	-	-
Microbacterium	1 [35]	0	1 [45]	1 [70]
Micrococcus	1 [35]	0	-	-
Mitsuokella	-	-	1 [50]	1 [67]
Mogibacterium	1[36]	2 [57,61]	-	-
Morganella	-	-	1 [50]	1 [44]
Mycobacterium	1 [35]	0	-	-
Negativibacillus	-	-	1 [50]	0
Neisseria	2 [35,68]	0	1 [52]	0
Nocardioides	-	-	0	1 [70]
Ochrobactrum	1 [35]	0	1 [40]	0
Odoribacter	0	1 [35]	0	1 [42]
Oribacterium	-	-	0	1 [70]
Oscillibacter	0	1 [57]	2 [50,52]	0
Oscillospira	0	2 [35,59]	-	-
Oxalobacter	-	-	1 [62]	0
Paenibacillus	0	1 [36]	-	-
Paeniclostridium	-	-	1 [54]	0
Pantoea	-	-	1 [44]	0
Papillibacter	0	1 [35]	-	-
Parabacteroides	0	1 [35]	9 [37,40,42,44,46,53,60,62,70]	0
Paracoccus	1 [35]	0	-	-
Paraprevotella	-	-	2 [40,42]	0
Parascardovia	0	1 [33]	-	-
Parvimonas	0	4 [57,59,61,68]	0	8 [37,42,44,59,61,63,67,70]
Pedobacter	2 [35,58]	0	-	-
Peptoniphilus	-	-	0	1 [64]
Peptococcus	-	-	0	1 [70]

	Peptostreptococcus	0	5 [57-59,61,68]	0	6 [42,44,54,59,63,67]
	Phascolarctobacterium	2 [36,68]	2 [35,57]	1 [40]	0
	Phenylobacterium	1 [35]	0	-	-
	Phyllobacterium	1 [35]	0	1 [70]	0
	Porphyromonas	0	2 [33,57]	0	1 [44]
	Portiera	-	-	0	1 [39]
	Prevotella	2 [35,36]	3 [57,59,68]	5 [42,46,50,54,67]	2 [44,58]
	Propionibacterium	3 [35,58,68]	0	1 [44]	0
	Providentia	-	-	0	1 [39]
	Pseudocalvibacter	1 [35]	0	-	-
	Pseudoflavonifractor	-	-	1 [50]	0
	Pseudomonas	2 [35,58]	1 [57]	5 [42,44,50,58,70]	0
	Pseudoramibacter_Eubacterium	0	1 [35]	-	-
	Pseudoxanthomonas	1 [35]	0	-	-
	Psychrobacter	-	-	1 [70]	0
	Ralstonia	1 [35]	0	-	-
	Rahnella	1 [58]	0	-	-
	Rhodococcus	1 [35]	0	1 [50]	0
	Roseburia	0	1 [35]	1 [46]	1 [45]
	Rubrobacter	1 [35]	0	-	-
	Ruminiclostridium	-	-	0	1 [44]
	Ruminococcus	1 [35]	1 [57]	6 [37,42,53,62,66,67]	0
	Salinispora	0	1 [35]	-	-
	Schwartzia	0	2 [61,68]	0	1 [61]
	Selenomonas	0	2 [61,68]	0	4 [44,62,67,70]
	Serratia	1 [36]	0	-	-
	Shewanella	0	1 [61]	0	2 [54,70]
	Sphingobacterium	1[58]	0	-	-
	Sphingobium	1 [35]	0	1 [67]	0
	Sphingomonas	2 [35,58]	0	2 [52,70]	0
	Staphylococcus	1 [35]	2 [33,57]	-	-
	Stenotrophomonas	2 [35,58]	0	0	1 [70]
	Streptococcus	0	3 [33,57,61]	0	7 [37,48,53,58,60,61,67]
	Streptomyces	1 [61]	0	1 [61]	0
	Streptophyla	0	1 [33]	-	-
	Streptophyta	1 [35]	0	-	-
	Succinivibrio	-	-	1 [50]	0
	Sutterella	1 [68]	0	1 [62]	0
	Treponema	-	-	1 [42]	2 [62,67]
	Turicibacter	-	-	1 [64]	0
	Veillonella	0	1 [33]	2 [54,67]	0
	Victivallis	0	1 [57]	-	-
**Species**	*Acinetobacter junii*	-	-	0	1 [52]
	Acinetobacter johnsonii	-	-	1 [70]	0
	Aggregatibacter segmis	0	1 [59]	-	-
	Akkermansia muciniphila	0	1 [68]	-	-
	Alcaligenes faecalis	1 [35]	0	-	-
	Atopobium parvulum	1 [68]	0	-	-
	Bacillus cereus	1 [35]	0	-	-
	Bacteroides acidifaciens	-	-	1 [70]	0
	Bacteroides eggerthii	-	-	1 [54]	0

Bacteroides fragilis	0	4 [35,59,65,68]	0	3 [43,52,59]
Bacteroides caccae	-	-	1 [46]	0
Bacteroides ovatus	0	1 [59]	1 [46]	0
Bacteroides uniformis	0	1 [35]	4 [39,46,60,70]	1 [52]
Bacteroides vulgatus	1 [65]	0	-	-
Blautia producta	1 [36]	0	-	-
Blautia wexlerae	-	-	1 [52]	0
Campylobacter ureolyticus	0	1 [59]	0	1 [70]
Catenibacterium mitsuokai	-	-	0	1 [52]
Christensenella timonensis	0	1 [68]	-	-
Clostridium aldenense	-	-	0	1 [70]
Clostridium hathewayi	-	-	0	1 [70]
Clostridium hiranonis	1 [36]	0	-	-
Clostridium sensu stricto	-	-	1 [44]	0
Clostridium oroticum	1 [68]	0	-	-
Clostridium perfringens	-	-	1 [54]	0
Clostridium ramosum	-	-	1 [70]	0
Dialister invisius	-	-	0	1 [52]
Erysipelotrichaceae incertae sedis	1 [57]	0	-	-
Erwinia dispersa	-	-	1 [46]	0
Escherichia coli	1 [59]	1 [65]	-	-
Eubacterium coprostanoligenes	0	1 [68]	-	-
Eubacterium eligens	-	-	1 [56]	0
Eubacterium sulci	-	-	0	1 [52]
Eubacterium ventriosum	-	-	1 [56]	0
Faecalibacterium prausnitzii	1 [59]	0	3 [39,46,60]	0
Fusobacterium nucleatum	0	2 [65,68]	0	3 [37,43,56]
Fusobacterium periodonticum	-	-	0	1 [52]
Gemella haemolysans	0	1 [65]	-	-
Gemella morbillorum	0	1 [68]	0	2 [43,52]
Haemophilus parainfluenzae	2 [65,68]	0	-	-
Intestinimonas butyriciproducens	0	1 [68]	-	-
Kocuria palustris	-	-	1 [52]	0
Lachnoanaerobaculum orale	-	-	0	1 [70]
Lachnospiracea incertae sedis	1 [57]	0	-	-
Lachnoclostridium citroniae	-	-	0	1 [52]
Lactobacillus brevis	-	-	1 [70]	0
Lactobacillus delbruecki	1 [35]	0	-	-
Leuconostoc gelidum	-	-	0	1 [52]
Methylobacterium adhaesivum	-	-	0	1 [70]
Parabacteroides distasonis	-	-	2 [46,70]	0
Parabacteroides merdae	-	-	0	1 [52]
Parvimonas micra	0	1 [68]	0	2 [43,52]
Peptostreptococcus micros	0	1 [35]	-	-
Peptostreptococcus stomatis	0	1 [68]	0	1 [43]
Porphyromonas endodontalis	0	1 [59]	-	-
Prevotella copri	1 [36]	0	1 [46]	0
Prevotella intermedia	0	1 [59]	-	-
Prevotella stercorea	1 [36]	0	-	-

Pseudomonas veroni	1 [59]	0	2 [59,70]	0
Psychrobacter sanguinis	-	-	1 [70]	0
Ralstonia mannitolilytica	-	-	1 [52]	0
Ruminococcus bromii	0	1 [68]	1 [70]	0
Ruminococcus callidus	0	1 [68]	-	-
Ruminococcus torques	1 [65]	0	-	-
Shewanella algae	-	-	0	1 [70]
Solobacterium moorei	0	1 [68]	-	-
Streptococcus bovis	-	-	0	1 [52]
Streptococcus intermedius	0	1 [65]	-	-
Streptococcus salivarius	-	-	0	1 [56]
Vibrio cholerae	1 [59]	0	-	0

Abbreviations: CRC: colorectal cancer; HC: healthy controls; NCT: non-cancerous tissue.

Supplementary Table S6. Qualitative synthesis showing the strong and the suggestive microbial taxonomic associations with CRC.

	Microbial taxa	CRC vs HC N (%) studies	CRC vs NCT N (%) studies
**Phylum**	Actinobacteria	2 (20%)	4 (14%)
	Bacteroidetes		5 (17%)
	Cyanobacteria		2 (7%)
	Fusobacteria	4 (40%)	8 (28%)
**Class**	Alphaproteobacteria	2 (20%)	
	Bacilli		2 (7%)
	Bacteroidia		2 (7%)
**Order**	Bacteroidales		2 (7%)
	Bifidobacteriales		2 (7%)
	Burkholderiales		2 (7%)
	Campylobacteriales		2 (7%)
	Clostridiales	2 (20%)	2 (7%)
	Fusobacteriales		2 (7%)
**Family**	*Bifidobacteriaceae*		2 (7%)
	Campylobacteriaceae		2 (7%)
	Fusobacteriaceae		3 (10%)
	Gemelllaceae		2 (7%)
	Muribaculaceae		2 (7%)
	Porphyromonadaceae		4 (14%)
	Rikenellaceae		4 (14%)
	Ruminococcaceae		7 (24%)
	Streptococcaceae		2 (7%)
**Genus**	*Acinetobacter*	2 (20%)	3 (10%)
	Akkermansia		3 (10%)
	Bacillus		3 (10%)
	Bifidobacterium		3 (10%)
	Blautia	3 (25%)	4 (14%)
	Bradyrhizobium		2 (7%)
	Brevundimonas	2 (20%)	
	Campylobacter	4 (40%)	9 (31%)
	Collinsella		3 (10%)
	Dehalobacterium	2 (20%)	
	Eikenella		2 (7%)
	Escherichia-Shigella	2 (20%)	
	Faecalibacterium	2 (20%)	5 (17%)
	Fusobacterium	6 (60%)	19 (66%)
	Gemella		3 (10%)
	Granulicatella	3 (30%)	3 (10%)
	Klebsiella	3 (30%)	
	Lachnoanaerobaculum		2 (7%)
	Megasphaera		2 (7%)
	Methylobacterium		2 (7%)
	Neisseria	2 (20%)	
	Oscillibacter		2 (7%)
	Oscillospira	2 (20%)	
	Parabacteroides		9 (31%)
	Paraprevotella		2 (7%)
	Parvimonas	4 (40%)	8 (28%)
	Pedobacter	2 (20%)	
	Peptostreptococcus	5 (50%)	6 (21%)
	Porphyromonas	2 (20%)	

	Propionibacterium	3 (30%)	
	Pseudomonas		5 (17%)
	Ruminococcus		6 (21%)
	Schwartzia	2 (20%)	
	Selenomonas	2 (20%)	4 (14%)
	Shewanella		2 (7%)
	Sphingomonas	2 (20%)	2 (7%)
	Stenotrophomonas	2 (20%)	
	Streptococcus	3 (30%)	7 (24%)
	Veillonella		2 (7%)
**Species**	*Bacteroides fragilis*	4 (40%)	3 (10%)
	Faecalibacterium prausnitzii		3 (10%)
	Fusobacterium nucleatum	2 (20%)	3 (10%)
	Gemella morbillorum		2 (7%)
	Haemophilus parainfluenzae	2 (20%)	
	Parabacteroides distasonis		2 (7%)
	Parvimonas micra		2 (7%)
	Pseudomonas veroni		2 (7%)

Strong positive association (≥3 studies in same direction; none in opposite direction);
Suggestive positive association (2 studies in same direction; none in opposite direction);
Strong negative association (≥3 studies in same direction; none in opposite direction);
Suggestive negative association (2 studies in same direction; none in opposite direction).
